# Ecological dynamics imposes fundamental challenges in community‐based microbial source tracking

**DOI:** 10.1002/imt2.75

**Published:** 2023-01-05

**Authors:** Xu‐Wen Wang, Lu Wu, Lei Dai, Xiaole Yin, Tong Zhang, Scott T. Weiss, Yang‐Yu Liu

**Affiliations:** ^1^ Channing Division of Network Medicine, Department of Medicine Brigham and Women's Hospital and Harvard Medical School Boston Massachusetts USA; ^2^ CAS Key Laboratory of Quantitative Engineering Biology Shenzhen Institute of Synthetic Biology, Shenzhen Institute of Advanced Technology, Chinese Academy of Sciences Shenzhen China; ^3^ University of Chinese Academy of Sciences Beijing China; ^4^ Environmental Microbiome Engineering and Biotechnology Laboratory, Department of Civil Engineering The University of Hong Kong Hong Kong China; ^5^ Center for Artificial Intelligence and Modeling, The Carl R. Woese Institute for Genomic Biology University of Illinois at Urbana‐Champaign Urbana Illinois USA

**Keywords:** microbial interactions, microbial source tracking, priority effects

## Abstract

Quantifying the contributions of possible environmental sources (“sources”) to a specific microbial community (“sink”) is a classical problem in microbiology known as microbial source tracking (MST). Solving the MST problem will not only help us understand how microbial communities were formed, but also have far‐reaching applications in pollution control, public health, and forensics. MST methods generally fall into two categories: target‐based methods (focusing on the detection of source‐specific indicator species or chemicals); and community‐based methods (using community structure to measure similarity between sink samples and potential source environments). As next‐generation sequencing becomes a standard community‐assessment method in microbiology, numerous community‐based computational methods, referred to as MST solvers hereafter have been developed and applied to various real datasets to demonstrate their utility across different contexts. Yet, those MST solvers do not consider microbial interactions and priority effects in microbial communities. Here, we revisit the performance of several representative MST solvers. We show compelling evidence that solving the MST problem using existing MST solvers is impractical when ecological dynamics plays a role in community assembly. In particular, we clearly demonstrate that the presence of either microbial interactions or priority effects will render the MST problem mathematically unsolvable for MST solvers. We further analyze data from fecal microbiota transplantation studies, finding that the state‐of‐the‐art MST solvers fail to identify donors for most of the recipients. Finally, we perform community coalescence experiments to demonstrate that the state‐of‐the‐art MST solvers fail to identify the sources for most of the sinks. Our findings suggest that ecological dynamics imposes fundamental challenges in MST. Interpretation of results of existing MST solvers should be done cautiously.

## Highlights

- Solving the microbial source tracking (MST) problem using existing MST solvers is impractical when ecological dynamics plays a role in community assembly.
- The presence of either microbial interactions or priority effects will render the MST problem mathematically unsolvable for MST solver.
- Community coalescence experiments show that MST solvers fail to identify the sources for most of the sinks.

## Graphical abstract

Quantifying the contributions of possible environmental sources (“sources”) to a specific microbial community (“sink”) is a classical problem in microbiology known as microbial source tracking (MST). We show compelling evidence that solving the MST problem using existing MST solvers is impractical when ecological dynamics plays a role in community assembly. In particular, we clearly demonstrate that the presence of either microbial interactions or priority effects will render the MST problem mathematically unsolvable for MST solvers.

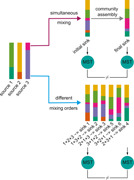

## INTRODUCTION

Estimating the contributions or mixing proportions of different source microbial communities (“sources”) to a specific microbial community (“sink”) is known as the microbial source tracking (MST) problem [1–3]. Historically, MST was framed in the context of quantifying the input of various sources of fecal contamination to manage and remediate water pollution [4]. Traditional MST methods are largely target‐based [1, 3], that is, focusing on the detection of predetermined source‐specific indicator species (e.g., human‐associated HF183 bacterial cluster) [5] or chemicals (e.g., cholesterol and coprostanol, which were usually classified as chemical source tracking (CST)) [6]. Recently, MST has been used in many other contexts, such as healthcare [7, 8] and forensics [9]. This is largely due to the advances in culture‐independent metagenomics and next‐generation sequencing technologies, which have enabled us to perform community assessment at an unprecedented speed [10–13]. Consequently, many community‐based computational methods, referred to as MST solvers, hereafter, have been developed to solve the MST problem by using community structure to measure the similarity between sink samples and potential source environments [2, 4].

MST solvers typically formalize the MST problem as follows. Consider a sink community represented by a composition vector x, where $x_{j}$ corresponds to the relative abundance of species‐j, 1≤j≤N. Let K be the number of known sources to this sink community. Each known source is represented by a composition vector $y^{\left(a\right)}$, where $y_{j}^{\left(a\right)}$ is the relative abundance of species‐*j* in source‐a(1≤a≤K). In addition to the K known sources, we assume there is an unobserved source labeled as (K+1). Our goal is to estimate the contributions or mixing proportions of the (K+1) source communities to form the sink community, i.e., inferring $m_{a}$ (a=1,…,K+1) that satisfy $\sum_{a=1}^{K+1}m_{a}y^{\left(a\right)}=x$ and $\sum_{a=1}^{K+1}m_{a}=1$.

Here, we introduce three representative MST solvers. The first solver is based on the classification analysis in machine learning, for example, using the random forest (RF) classifier [14]. In this case, each source represents a distinct class, and RF will classify the sink into different classes with different probabilities. The probabilities of the sink belonging to the different classes can be naturally interpreted as the mixing proportions or contributions of those sources to the sink. Beyond the simple classification analysis, more advanced statistical methods based on Bayesian modeling have been developed. For example, SourceTracker is a Bayesian MST solver that explicitly models the sink as a convex mixture of sources and infers the mixing proportions via Gibbs sampling [15]. FEAST (fast expectation‐maximization for microbial source tracking [16]) is a more recent statistical method. FEAST also assumes each sink is a convex combination of sources. But it infers the model parameters via fast expectation‐maximization, which is much more scalable than Markov Chain Monte Carlo used by SourceTracker.

Both SourceTracker and FEAST have shown promising performance in synthetic datasets and offered biologically meaningful interpretations when applied to real datasets under certain contexts. Yet, the synthetic datasets used to validate these MST solvers were all generated from statistical distributions, rather than dynamics models in community ecology. Hence, the ecological dynamics driving the community assembly is completely ignored. We hypothesize that, after considering the ecological dynamics, the power of those MST solvers might be significantly restricted. The interpretation of their results should be done cautiously.

**Figure 1:**
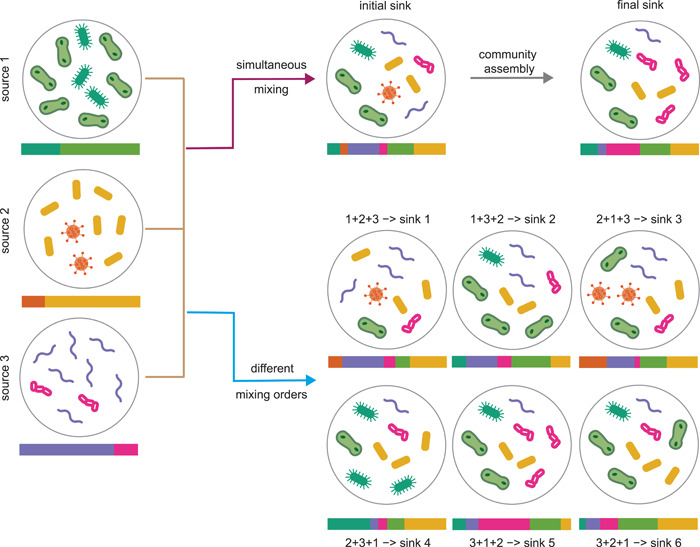
Ecological dynamics imposes fundamental challenges in microbial source tracking. (Top) A sink is obtained by simultaneously mixing three sources (without any species overlap) with mixing proportions (1/3, 1/3, 1/3). Due to the presence of microbial interactions, the initial composition of the sink community (right after the mixing, which is typically not available for microbial source tracking [MST]) can be significantly different from the final composition (which is the input of MST solvers). Applying any MST solver to the final sink composition will yield different results from applying the MST solver to the initial sink composition. (Bottom) Due to the priority effects, three sources mixed with different orders can result in a total 3!=6 different sinks with different compositions, even if the mixing proportions of the sources are exactly the same for the different mixing orders.

Here, we consider two factors that heavily affect the ecological dynamics and community assembly: (1) microbial interactions; (2) priority effects. Microbial interactions are ubiquitous. They can be mediated by direct secretion of substances such as bacteriocins [17, 18], ecological competition between the microbes [19], metabolite exchange [20], or the host's immune system modulation [21–23]. In the presence of microbial interactions, the final composition of the sink community will, in general, be fundamentally different from its initial one, that is, the one right after the source mixing, which is typically not available to us (see Figure 1). Consequently, the source contributions (or mixing proportions) estimated by applying MST solvers to the final sink community will be significantly different from the source contributions estimated by applying MST solvers to the initial sink community.

Ecological theory suggests that the establishment of new species in a community can depend on the order and/or timing of their arrival, a phenomenon known as *priority effects* [24–27]. This phenomenon is actually ubiquitous in animal [28, 29], plant [30], and microbial communities [24, 31, 32]. Mechanisms of priority effects and evidence for their importance have been heavily studied for microbial communities inhabiting a range of environments, including the mammalian gut [33–36], the plant phyllosphere [37–39], and rhizosphere [40, 41], soil [42], freshwater, [43] and oceans [44, 45]. For example, it has been pointed out that priority effects probably shape the human gut microbiome during early childhood [46]. In particular, the infant's exposure history and the patterns of dispersal from various sites in or on their mother could mediate the observed mutual exclusion between *Bacteroides* spp., *Escherichia* spp. and lactic acid producers such as *Bifidobacterium* spp. and *Lactobacillus* spp. [46]. In the presence of priority effects, even if the mixing proportions (source contributions) are exactly the same, sink communities resulting from mixing the same set of sources, but with different mixing orders could be drastically different (see Figure 1). Thus, for the different sink communities, the source contributions estimated by MST solvers will also be quite different, contradicting the truth.

To test our hypothesis, in this work, we first systematically examined the impact of microbial interactions and priority effects on the performance of existing MST solvers using synthetic data generated by a classical population dynamics model in community ecology. We found that those solvers fail in the presence of microbial interactions or priority effects. We offered mathematical explanations for the failures. We then applied FEAST and SourceTracker, the two state‐of‐the‐art MST solvers, to analyze data from two fecal microbiota transplantation (FMT) studies, finding that they fail to identify donors for most of the recipients. To experimentally validate our hypothesis, we performed community coalescence experiments, where fecal samples from 24 healthy individuals (i.e., sources) were mixed and cultured ex vivo to form 481 sink communities. We found that FEAST and SourceTracker fail to identify sources for most of the sinks. These results underscore the fundamental challenges imposed by ecological dynamics in solving the MST problem using computational approaches.

## RESULTS

### Impact of microbial interactions on community‐based MST

To illustrate the impact of microbial interactions on community‐based MST, we simulated source and sink communities as the steady states of a classical population dynamics model in community ecology—the Generalized Lotka‐Volterra (GLV) model: $dX_{i}/dt=X_{i}\left(r_{i}+\sum_{j=1}^{N}a_{\mathrm{ij}}X_{j}\right),i=1,\text{…},N$. Here, $X_{i}$ is the abundance (or biomass) of species‐i, and $r_{i}$ is its intrinsic growth rate. The microbial interaction matrix $A=\left(a_{\mathrm{ij}}\right)\in R^{N\times N}$ can be represented by an ecological network G(A): there is a directed edge (j→i) in the network if and only if $a_{\mathrm{ij}}\neq 0$. And $a_{\mathrm{ij}}>0$ (<0, or =0) means that species‐j promotes (inhibits or does not affect) the growth of species‐*i*, respectively. To generate the matrix A, we first generate the underlying network G(A) using a random graph model [47] with N nodes (species) and connectivity C (representing the probability of randomly connecting two nodes). Then for each link (j→i)∈G(A) with j≠i, we draw $a_{\mathrm{ij}}$ from a normal distribution $N(0,\sigma^{2})$. Here, the standard deviation σ of this normal distribution can be considered as the characteristic inter‐species interaction strength. Despite its simplicity, the GLV model has been successfully applied to describe the population dynamics of various microbial communities, from the soil [48] and lakes [49] to the human gut [50, 51].

We generated three source communities, $S_{1}$, $S_{2}$, and $S_{3}$, each with 30 species drawn from a pool of N=90 species. To simplify the MST problem, we ensured the three sources do not share any common species, and the intrinsic growth rates of all species were set to be identical ($r_{i}=0.5$ for i=1,…,N). The composition vectors of $S_{1}$, $S_{2}$, and $S_{3}$ (denoted as $y^{\left(1\right)},y^{\left(2\right)},y^{\left(3\right)}$, respectively) were obtained by running the GLV model until a steady state was reached and then normalizing the steady‐state abundance of each species by the total biomass of the community (see Supporting Information: Section 1 for details).

To systematically examine the impact of microbial interactions on community‐based MST, we tuned the connectivity C of the ecological network G(A) and the characteristic inter‐species interaction strength σ in the GLV model. For a given pair of (C,σ), we simulated 100 sink communities with the initial composition vector x(0) given by a random mixture of the three source communities, that is, $x(0)=m_{1}y^{\left(1\right)}+m_{2}y^{\left(2\right)}+m_{3}y^{\left(3\right)}$, where $m_{a}$'s were drawn from uniform distribution U(0,1) with the constraint that $\sum_{a}m_{a}=1$. The final composition of each sink was obtained by running the GLV model until a steady state. Note that to disentangle the impacts of microbial interactions and priority effects on community‐based MST, here we assume a simultaneous mixing, that is, all the sources (and their species) are available at the same time to avoid priority effects.

**Figure 2:**
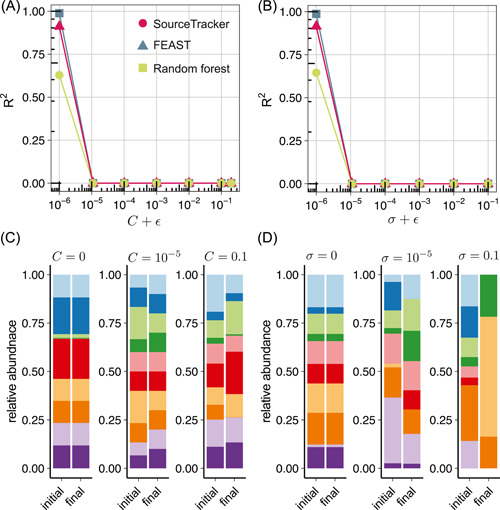
Impact of microbial interactions on microbial source tracking (MST). (A, B) Performance of SourceTracker (red), fast expectation‐maximization for microbial source tracking (FEAST) (blue), and random forest (green) in simulated sinks with different network connectivity C (A) and characteristic interaction strengths σ (B). Each simulation was performed using three synthetic sources and 100 synthetic sinks. Accuracy of each method is measured as the coefficients of determination ($R^{2}$) of the estimated proportions. Each point represents the mean $R^{2}$ for three independent source sets; error bars show s.e.m (n=3) of the mean of $R^{2}$. (C, D) Initial and final steady compositions (we only show the relative abundance of the first 10 species for visualization purposes) of a sink with different network connectivity (C) and characteristic interaction strengths (D). In (A, C), the diagonal elements of the interaction matrix A are set to be $a_{\mathrm{ii}}=-5C$ to ensure the stability of the community, and the characteristic interaction strength σ=0.1. In (B, D), we set $a_{\mathrm{ii}}=-5\sigma$ to ensure stability and network connectivity C=0.5. In all the simulations, we set the intrinsic growth rate r=0.5 for all the species. We added a pseudo number ${\epsilon =10}^{-6}$ to the *x*‐axis for visualization purposes.

We found that, with identical intrinsic species growth rates, both FEAST and SourceTracker can achieve very high performance (with the coefficients of determination of the estimated proportions $R^{2}=1$) in the absence of microbial interactions: C=0 (Figure 2A) or σ=0 (Figure 2B). This can be explained as follows. First, in the absence of microbial interactions and with identical intrinsic species growth rates, the final composition of each sink will be identical to its initial composition (right after the mixture of the three sources). Second, the three sources do not share any common species, hence the MST problem becomes trivial for those solvers that assume each sink is a convex combination of sources. Note that even in this ideal case, the classification‐based MST solver (i.e., RF) does not perform very well. This is because, as the combination of different sources, the sink community's composition does not necessarily need to be similar to the composition of any source.

Interestingly, with a nonzero C or σ, none of the three MST solvers can successfully estimate the source contributions (indicated by $R^{2}\approx 0$). This implies that the existing MST solvers will completely fail as long as microbial interactions are present, and even in the absence of priority effects (see Figure 2A,B).

The insolvability of the MST problem in the presence of microbial interactions can be conceptually explained as follows. Any microbial interactions will drive the sink community to evolve from its initial state to its final state (Figure 2C,D). The final state will be generally different from the initial one. There are two exceptions. First, the initial sink community is already at its steady state and hence will not change over time. This case almost never happens, because the initial sink is obtained by mixing multiple sources. Even though the sources are at their respective steady states, simply mixing them will not lead to another steady state. The interactions among the species across different sources will affect the assembly of the sink community. Some source‐specific species might even die out due to competition. Second, the system has a periodic trajectory in the state space, and the initial and final states happen to be identical. This coincidence generally will not happen for an unspecific time interval between the initial and final states (see Supporting Information: Section 2 for a more mathematical explanation on the difference between the initial and final states of the sink community, using generic population dynamics models). Since the initial and final states of the sink community are different, the source contributions estimated by applying any MST solver to the final sink community will also be different from that estimated by applying the MST solver to the initial sink community. We can avoid this issue by inferring the initial state from the final state. But this is impossible if the system is globally stable, that is, any feasible initial state will result in the same final state. Even if such global stability does not exist, inferring the initial state from the final one would typically require detailed knowledge of the ecological dynamics, which is not known a priori. All these factors suggest that without prior knowledge of the ecological dynamics, the MST problem is mathematically unsolvable in the presence of microbial interactions. Numerous computational methods have been developed to infer the ecological dynamics of microbial communities from either temporal or steady‐state data [51–53]. Yet, those methods typically require high‐quality absolute abundance data. Moreover, the performance of those methods (especially those relying on temporal data) can be largely affected by model misspecification. In principle, we can use symbolic regression techniques to infer both the model structure and parameters from temporal data [54–58]. But this also requires highly informative temporal data, which is not readily available for complex microbial communities (e.g., the human gut microbiome.

In the extreme case, for instance, the growth rate of each species is extremely low, and inter‐species interactions are rare and weak, existing MST solvers can identify the true source with reasonable accuracy, but estimated source contributions might be quite inaccurate, rendering a low $R^{2}$ between the true and estimated source contributions (see Supporting Information: Figure S1). Moreover, there exists a characteristic time scale which is the time interval between the initial sink community and sink community sequenced for MST. We found that before the characteristic time scale, existing community‐based MST solvers, for example, FEAST works well, and this time scale certainly depends on the network connectivity, interaction strength, and mean of species’ growth rate (see Supporting Information: Figure S2).

### Impact of priority effects on community‐based MST

**Figure 3:**
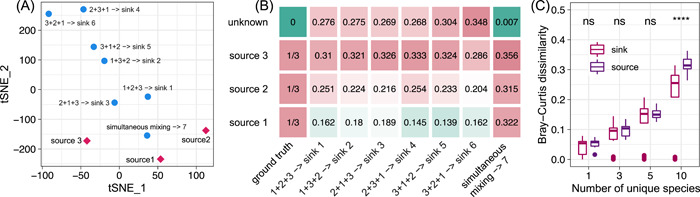
Impact of priority effects on microbial source tracking (MST). (A, B) We synthesized three sources, $S_{1}$, $S_{2}$, and $S_{3}$, whose species collections do not have any overlap (30 species for each source). We mixed these three sources using six different mixing orders but with the same mixing proportions $\left(\frac{1}{3},\frac{1}{3},\frac{1}{3}\right)$, rendering six sinks. We set the network connectivity C=0.5, the characteristic interaction strength σ=1, and the intrinsic growth rate r=0.5 for each species. We set the diagonal elements of interaction matrix A to be $a_{\mathrm{ii}}=-5$ to ensure stability. (A) Dimensionality reduction using t‐SNE shows the variations among the six sinks generated from the six different mixing orders. (B) Contribution of each source to the six simulated sinks estimated by fast expectation‐maximization for microbial source tracking (FEAST). (C) Between‐sink and between‐source Bray‐Curtis dissimilarity. We synthesized five sources. The species collection of each source includes $N_{u}$ unique species and the remaining $\left(90-5N_{u}\right)$ species are shared by all the sources. We mixed these five sources with the same mixing proportions $\left(\frac{1}{5},\frac{1}{5},\frac{1}{5},\frac{1}{5},\frac{1}{5}\right)$ in 100 different mixing orders randomly chosen from the total 5!=120 mixing orders. We set the network connectivity C=0.5, the characteristic interaction strength σ=1, and the intrinsic growth rate r=0.5 for each species. We set the diagonal elements of interaction matrix A to be $a_{\mathrm{ii}}=-10$ to ensure stability. *p*‐values were calculated using one‐sided Wilcoxon test.

To examine the impact of priority effects on community‐based MST, we again simulated three source communities, $S_{1}$, $S_{2}$, and $S_{3}$, whose species collections do not have any overlap (30 species for each source). The final compositions of sources were obtained by running the GLV model until reaching a steady state and then normalizing the steady‐state abundance of each species by the total biomass of the community (see Supporting Information: Section 1 for details). For each of the 3!=6 mixing orders, we generated a sink by mixing three sources with equal proportions $\left(\frac{1}{3},\frac{1}{3},\frac{1}{3}\right)$, then ran the GLV model to obtain its final composition. For comparison purposes, we also generated a sink through the simultaneous mixing of the three sources with equal proportions $\left(\frac{1}{3},\frac{1}{3},\frac{1}{3}\right)$. We visualized the compositions of the three sources and the seven sinks using the t‐distributed stochastic neighbor embedding (t‐SNE) method, finding that the compositions of the seven sinks are clearly different (see Figure 3A). We then ran FEAST, the fastest MST solver, to estimate the contributions of the three sources to each sink, finding that the contributions are different for different sinks, despite the true mixing proportions being exactly the same (Figure 3B). In the above simulations, we set the network connectivity C=0.5 and the characteristic interaction strength σ=1.

The above results make us wonder about the solvability of the community‐based MST problem in the presence of priority effects. Here, we offer an outline of proof that community‐based MST is mathematically unsolvable in the presence of priority effects. Consider a set of source communities. If we mix them in different orders (but using the same set of mixing proportions), this will generally lead to different sink communities due to priority effects. For example, we synthesized five sources. The species collection of each source includes $N_{u}$ unique species, and the remaining $\left(90-5N_{u}\right)$ species are shared by all the sources. We mixed these five sources with the same mixing proportions $\left(\frac{1}{5},\frac{1}{5},\frac{1}{5},\frac{1}{5},\frac{1}{5}\right)$ in 100 different mixing orders randomly chosen from the total 5!=120 mixing orders. We found that the between‐sink dissimilarity can be as large as the between‐source dissimilarity (see Figure 3C). We emphasize that different mixing orders generally result in different sink communities even in the absence of any microbial interactions (see Supporting Information: Section 3 for a mathematical explanation). For different sink communities, the source contributions estimated by any computational method (i.e., MST solver) will also be different, contradicting the fact that the source contributions (i.e., mixing proportions) are exactly the same. This proof by contradiction clearly illustrates that community‐based MST is mathematically unsolvable in the presence of priority effects.

### Evaluation of MST solvers using data from FMT studies

During FMT, fecal microbiota from a carefully screened, healthy donor is introduced to a recipient through either the lower or upper gastrointestinal tract. It is a “natural” mixing experiment that can be used to evaluate the performance of MST solvers. To achieve that, we applied FEAST and SourceTracker to analyze data from two FMT studies [59, 60].

**Figure 4:**
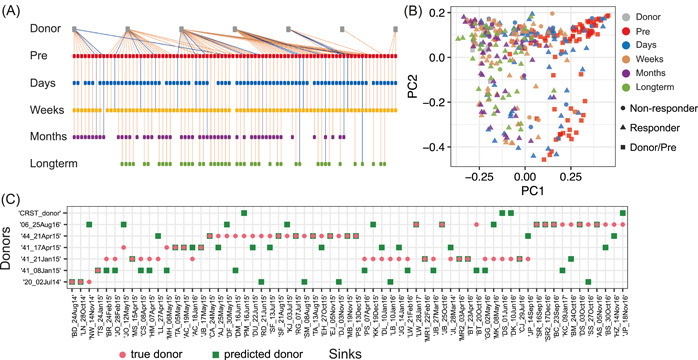
Evaluation of fast expectation‐maximization for microbial source tracking (FEAST) using fecal microbiota transplantation (FMT) data from Staley et al. (A) Donor‐recipient relationship. Each trajectory represents a donor and its corresponding recipients at up to five time points. Trajectories of recipients who responded to FMT (i.e., responders) are colored in yellow. Trajectories of nonresponders are colored in blue. (B) Principal coordinates analysis (PCoA) plot based on the Bray‐Curtis dissimilarity. (C) True donor (red cycle) versus predicted donor (green square) of each recipient. For each post‐FMT community (sink), among all the seven donors, we referred to the one whose fecal sample has the highest contribution estimated by FEAST as the “predicted donor.” Here, we only showed the results for the first 65 sinks for visualization purposes (see Supporting Information: Figure S3 for results of the remaining 194 sinks). Sources: microbiome samples of donors and the pre‐FMT samples of recipients; Sinks: post‐FMT samples of recipients.

In the first study, recurrent *Clostridioides difficile* infection (rCDI) patients were treated with encapsulated donor material for FMT (cap‐FMT) [60]. Figure 4A shows the donor‐recipient relationship between seven healthy donors and 88 rCDI patients (i.e., recipients). Each trajectory represents a donor and one of its recipients with fecal samples collected at (up to) five different time points: pre‐FMT, 2–6 days post‐FMT, weeks (7–20 days) post‐FMT, months (21–60 days) post FMT, and long term (>60 days). The principal coordinate analysis (PCoA) plot of all the microbiome samples is shown in Figure 4B. We tested if FEAST can correctly identify the donor of a recipient. To achieve that, we considered each post‐FMT sample of each recipient as a sink community and considered the fecal samples of all seven donors, as well as the recipient's pre‐FMT sample, as potential source communities. Then we applied FEAST to solve the MST problem. For each sink community, among all the seven donors, we referred to the one whose fecal sample has the highest contribution estimated by FEAST as the “predicted donor” (green squares, Figure 4C, Supporting Information: Figure S3). Interestingly, we found that for a large portion (61%) of the sink communities, FEAST failed to identify the true donor (red circles, Figure 4C, Supporting Information: Figure S3), though the average Jensen‐Shannon divergence among those donors is higher enough (0.63). Similar results were found for SourceTracker (see Supporting Information: Figure S4). These results clearly demonstrate the limitation of existing MST solvers.

In the second FMT study, the gut microbiota of human donors with autism spectrum disorder (ASD) or typically‐developing (TD) controls were transplanted into germ‐free mice [59]. The data set includes eight donors, 13 recipients, and in total 106 post‐FMT sink communities. We again examined whether FEAST can correctly identify the true donor of each sink community. For each sink community, among the eight donors, we refer to the one whose fecal sample has the highest contribution predicted by FEAST as the “predicted donor” (green squares, Supporting Information: Figure S5). We found that for 40% of the sink communities, FEAST failed to identify the true donor (red circles, Supporting Information: Figure S5). Similar results were observed for SourceTracker (see Supporting Information: Figure S6).

### Evaluation of MST solvers using data from community coalescence experiments

**Figure 5:**
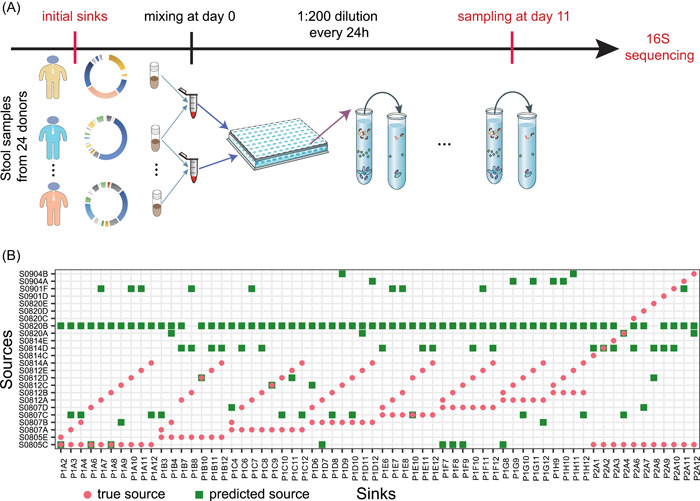
Evaluation of fast expectation‐maximization for microbial source tracking (FEAST) using data from pairwise community coalescence experiments. (A) Schematic diagram of the community coalescence experiments. There are 24 source communities (stool samples from 24 healthy individuals). Each sink community is obtained by mixing two different source communities ex vivo and the final composition of each sink was obtained from metagenomic sequencing of samples collected after 11 days of the ex vivo mixing. (B) True sources (red cycles) versus predicted sources (green squares) of each sink. For each sink, among the 24 known sources, the two sources with the top‐two largest contributions predicted by FEAST were referred to as the predicted sources. Here, we only showed the first 64 sinks for the visualization purpose (see Supporting Information: Figure S5 for results of the remaining 192 sinks).

To further evaluate MST solvers using real data, we performed community coalescence experiments, where fecal microbiota from 24 healthy individuals (i.e., sources) were mixed and cultured ex vivo to form 481 sink communities (see Supporting Information: Section 4 for details). Among the 481 sinks, 256 sinks were obtained by mixing two different sources (pair‐wise mixing), and the remaining 225 sinks were obtained by mixing four different sources (quadruple‐wise mixing). After inoculation, the sink communities were transferred into fresh medium every 24 h (1:200 dilution) for 10 transfers [61] (see Figure 5A). Samples collected at the final time point (after 11 days of ex vivo mixing) were sequenced, and the resulting taxonomic profiles were considered as the steady‐state composition of sinks (see Methods). As expected, we found that the source and sink communities had distinct taxonomic profiles (Supporting Information: Figures S7 and S8).

We first applied FEAST and SourceTracker into the samples collected in a small‐scale control experiment. This experiment includes the longitude samples of eight subjects collected after transferring for 1–10 days. Here, we considered the eight samples at Day 0 as the possible sources and the samples collected at each following transfer as sinks. We found that the accuracy of FEAST (SourceTracker) in identifying the true source for sinks on Day 1 is over 75% (63%). However, the accuracy of both solvers will decrease in source prediction for sinks collected after more transfers (see Supporting Information: Figure S9). Despite each source in this control experiment being without mixing, the growth rates for different species in a particular environment might be quite different, which also causes the initial sink community just after mixing to be different from the community for sequencing. This finding is consistent with our simulation in Supporting Information: Figure S1 that existing MST solvers can identify the true source with reasonable accuracy as long as the growth rate of each specie is extremely low, and inter‐species interactions are rare and weak.

To examine the performance of FEAST in community coalescence experiments, we applied FEAST to analyze the compositions of the 256 sinks obtained in the pair‐wise mixing experiments. We ranked the estimated contributions of 24 potential sources to each sink and selected the top‐two as the predicted sources. We found that the predicted sources (green squares) are different from the true sources (red circles) for most of the 256 sinks (Figure 5B and Supporting Information: Figure S10). This is also true for the cases of quadruple‐wise mixing (Supporting Information: Figure S12). Similar results were observed for SourceTracker (see Supporting Information: Figures S11 and S13).

Note that some donor samples (e.g., S0820B, S0814D) were predicted as sources for many sinks. We found this is due to the high abundance of common ASVs shared by sinks and those particular sources (Supporting Information: Figure S14).

## DISCUSSION

Many computational methods have been developed to solve the community‐based MST problem. Yet, those methods ignored the underlying ecological dynamics that drive the assembly of microbial communities. For example, as a Bayesian MST solver, SourceTracker explicitly models the sink as a convex mixture of sources and infers the mixing proportions via Gibbs sampling [15]. This approach was inspired by the “analogy” between quantifying the proportion of different source environments to a sink microbial community and inferring the mixing proportions of conversation topics in a test document [62, 63]. Here, we point out that this analogy is inappropriate. In topic modeling, which is a specific research area in natural language processing, the goal is to discover the abstract “topics” that occur in a collection of documents. In a sense, those documents are static or “dead.” By contrast, in MST, we are typically dealing with alive (or even flourishing) microbial communities, where ecological dynamics plays an important role in community assembly and determining their state, that is, the microbial composition. In the presence of ecological dynamics, a sink community cannot be simply considered a convex mixture of known and unknown sources. In this work, through numerical simulations, analytical calculations, and real data analysis, we presented compelling evidence that ecological dynamics impose fundamental challenges in community‐based MST. In particular, we clearly demonstrate that the presence of either microbial interactions or priority effects will render the community‐based MST problem mathematically unsolvable.

Existing MST solvers have been applied to various real datasets and demonstrated their utility across two fundamentally different contexts. First, as originally intended, they were used to quantify the contribution of different source environments to a sink microbial community. For example, SourceTracker was used to estimate the contributions of bacteria from “gut,” “oral,” “skin,” “soil,” and “unknown” sources to several indoor sink environments (e.g., office buildings, hospitals, and research laboratories) [15]. It was found that wet‐lab surface communities tended to be composed mainly of bacteria from “skin” and “unknown,” while neonatal intensive care units and office communities were typically dominated by skin bacteria. FEAST was used to estimate if taxa in the infant's gut originate from the birth canal, or if they are derived from some other external source at a later time point [16]. By treating samples taken from the infants at age 12 months as sinks, considering respective earlier time points and maternal samples as sources, a significantly larger maternal contribution in vaginally delivered infants over cesarean‐delivered infants was found. Moreover, biological mothers were more likely to be identified as sources of their infant's microbiome than other potential source communities. Although these results seem reasonable and agree well with our intuition, we suggest that the whole community of microbiome research should be very cautious when interpreting the results of existing MST solvers in this context. MST solvers might detect the sources with reasonable accuracy, but their estimated source contributions might be quite different from the true contributions due to complex ecological dynamics. This is particularly important for microbial communities living in nutrient‐rich environments such as the human gut. For microbial communities living in oligotrophic environments, the growth rates of bacteria and the assembly process of communities are relatively slow [64–66], if sources are from considerably different habitats (e.g., fecal samples from different animals) [67–69], and the impact of ecological dynamics on community‐based MST might be relatively low [70] (which is consistent with our numerical results shown in Figure S1). But even in this case, interpreting the results of existing MST solvers should be done with great caution.

Second, MST solvers have been used as a metric of similarity [16]. In this context, instead of quantifying the contribution of different sources to a sink, they aim for capturing the similarities between the sink and its characteristic environments using mixing proportions estimated by MST solvers. Each sink can be represented by a similarity feature vector, characterizing its similarity to each of its characteristic environments. For example, FEAST has been used in this context to distinguish patients in ICU from healthy adults, and capture shifts in microbial community composition that may underlie differences between pathogenic and neutral phenotypes [16]. We think this is a much more meaningful and practical way of using MST solvers to analyze real data.

## METHODS

### Community coalescence experiments

Stool samples from healthy human donors were collected and immediately transferred into the anaerobic workstation (85% N_2_, 10% H_2_, and 5% CO_2_, COY). Ten‐gram stool samples were suspended into 50 ml 20% glycerol (in sterile phosphate‐buffered saline, with 0.1% l‐cysteine hydrochloride). The samples were homogenized by vortexing and then filtered with sterile nylon mesh to remove large particles in fecal matter. Aliquots of the suspension were placed in sterile cryogenic vials and frozen at −80°C for long‐term storage until use.

Stool samples of 24 individuals were used for the community coalescence experiments. To generate 481 sink communities, samples from two, three, or four different individuals were mixed with equal volume. Twenty microliter stool mixture was inoculated into 980 μL medium in 96‐well plates (PCR‐96‐SG‐C, Axygen) for static culturing at 37°C in the anaerobic workstation. The medium used for ex vivo culture was modified from previous studies, which comprises: peptone water (2.0 g /L, CM0009, Thermo Fisher), yeast extract (2.0 g /L, LP0021B, Thermo Fisher), l‐cysteine hydrochloride (1 g/ L), Tween 80 (2 ml/L), hemin (5 mg/L), vitamin K1(10 μl/L), NaCl (1.0 g /L), K_2_HPO_4_ (0.4 g/L), KH_2_PO_4_ (0.4 g/L), MgSO_4_ ⋅ 7H_2_O (0.1 g/L), CaCl_2_ ⋅ 2H_2_O (0.1 g/L), NaHCO_3_ (4 g/L), porcine gastric mucin (4 g/L, M2378, Sigma‐Aldrich), sodium cholate (0.25 g/L) and sodium chenodeoxycholate (0.25 g/L) [71]. Ex vivo culture of gut microbial communities was transferred into fresh medium every 24 h (1:200 dilution), for a total of 10 transfers. After each transfer, samples were centrifuged to remove the supernatant, and the pellets were stored at −80°C with a plastic seal until DNA extraction.

The initial stool and ex vivo‐cultured samples after 10 passages were sequenced. For stool samples, DNA was extracted using the QIAamp Power Fecal Pro DNA Kit (Qiagen) according to the manufacturer's instructions. For cultured samples, DNA extraction (DNeasy UltraClean 96 Microbial Kit) and 16S amplicon library preparation were performed by an automated protocol at Tecan Freedom EVO 200. V3‐V4 region of the 16S rRNA gene was amplified using primers 341F 5′‐CCTACGGGNGGCWGCAG‐3′ and 805R 5′‐GACTACHVGGGTATCTAATCC‐3′ with custom barcodes [72]. Libraries were further pooled together at equal molar ratios and sequenced by Illumina NovaSeq (250 bp paired‐end reads) at Novogene Technology.

16S amplicon sequencing data were analyzed by QIIME2 (version 2020.2) [73]. Primers of the raw sequence data were cut with Cutadapt (via q2‐cutadapt) [74]. Quality control was performed by DADA2 (via q2‐dada2) [75]. All amplicon sequence variants (ASVs) from DADA2 were used to construct a phylogenic tree with fasttree2 (via q2‐phylogeny) [76]. The ASVs were assigned to taxonomy with naïve Bayes classifier (via q2‐feature‐classifier) [77] against the SILVA database (SILVA_132_SSURef_Nr99). The ASV table was normalized, and rare ASVs (all features with a total abundance of less than 10 and present in only a single sample) were filtered out.

### Evaluation of MST solvers

To evaluate MST solvers using simulated data, we generated 3 synthetic sources and 100 synthetic sinks. The performance of each method is measured as the coefficients of determination ($R^{2}$) of the estimated proportions with three independent source sets. For real datasets, we applied FEAST and SourceTracker to solve the MST problem. For each sink community, among all the possible sources, we referred to the one whose sample has the highest contribution estimated by FEAST (SourceTracker) as the predicted source.

## AUTHOR CONTRIBUTIONS

Yang‐Yu Liu conceived and designed the project. Xu‐Wen Wang and Yang‐Yu Liu did the analytical calculations. Xu‐Wen Wang did all the numerical calculations and analyzed all the simulated and real datasets. Lu Wu and Lei Dai designed and performed the community coalescence experiments. Xu‐Wen Wang and Yang‐Yu Liu wrote the manuscript. Lu Wu, Lei Dai, Xiaole Yin, Tong Zhang, and Scott T. Weiss interpreted the results, reviewed, and edited the manuscript. All authors approved the manuscript.

## CONFLICT OF INTEREST

The authors declare no conflict of interest.

## Supporting information

Supporting information.

## Data Availability

The sequencing data from the first FMT study is available at Sequence Read Archive at the National Center for Biotechnology Information under BioProject accession number SRP070464. The sequencing data from the second FMT study is available at Qiita [78] with ID: 11809. The raw sequencing data from the community coalescence experiments is available at European Nucleotide Archive (ENA) under study accession number PRJEB51290. The code used to generate the simulated data is available at: https://github.com/spxuw/MST. Supplementary materials (figures, tables, scripts, graphical abstract, slides, videos, Chinese translated version, and updated materials) may be found in the online DOI or iMeta Science http://www.imeta.science/
