# Correction to “ecological dynamics imposes fundamental challenges in community‐based microbial source tracking”

**DOI:** 10.1002/imt2.145

**Published:** 2023-10-19

In Wang et al. [1], the following ethics statement should have been added.

The ethics application (No. SIAT‐IRB‐200315‐H0438) was approved by the ethics committee of the Institutional Review Board of Shenzhen Institutes of Advanced Technology.

The authors apologize for the oversight.
