# Supplementary material for: Ecological dynamics imposes fundamental challenges in community‐based microbial source tracking
Source: Imeta. 2023 Jan 5;2(1):e75. doi: 10.1002/imt2.75 (PMC10989786; doi:10.1002/imt2.75)
Supplement: Supplementary file 1 — Supporting information. [file IMT2-2-e75-s001.pdf]

Ecological Dynamics Imposes Fundamental Challenges in Community-based Microbial Source Tracking

Supplementary Information

Xu-Wen Wang[1], Lu Wu[2], Lei Dai[2,3], Xiaole Yin[4], Tong Zhang[4], Scott T. Weiss[1] & Yang-Yu Liu[1]

[1]Channing Division of Network Medicine, Department of Medicine, Brigham and Women's Hospital and Harvard Medical School, Boston, MA, 02115, USA.

[2]CAS Key Laboratory of Quantitative Engineering Biology, Shenzhen Institute of Synthetic Biology, Shenzhen Institute of Advanced Technology, Shenzhen 518055, China.

[3]University of Chinese Academy of Sciences, Beijing 100049, China.

[4]Environmental Microbiome Engineering and Biotechnology Laboratory, Department of Civil Engineering, The University of Hong Kong, Hong Kong, China.

Table of Contents

1. Using an ecological model to generate synthetic microbiome data. 2

2. Microbial interactions affect the assembly of the sink community. 4

3. Priority effects affect the assembly of the sink community. 5

References... 6

1. Using an ecological model to generate synthetic microbiome data.

To systematically reveal the impacts of the microbial interactions and priority effects on MST, we generated synthetic data using the classical Generalized Lotka-Volterra (GLV) model[1]:

$$\frac{dX_i(t)}{dt} = X_i(t)\left[r_i + \sum_{j=1}^{N} a_{ij}X_j(t)\right], i = 1, \cdots, N.$$

Here $X_i(t)$ represents the absolute abundance of species-i at time $t \geq 0$, r_i is its intrinsic growth rate, which is randomly drawn from a uniform distribution $\mathcal{U}(0,1)$, if not specified otherwise. The inter-species interactions are encoded in the interaction matrix $\mathbf{A} = (a_{ij}) \in \mathbb{R}^{N\times N}$, with $a_{ij} > 0$ (< 0, or $= 0$) means that species-j promotes (inhibits or does not affect) the growth of species-i, respectively. To generate the matrix $\mathbf{A}$, we first generate the underlying ecological network $\mathcal{G}(\mathbf{A})$ using an Erdős-Rényi random graph model[2] with N nodes (species) and connectivity C (the probability of randomly connecting two nodes). Then for each link $(j \to i) \in \mathcal{G}(\mathbf{A})$ with $j \neq i$, we draw a_{ij} from a normal distribution $\mathbb{N}(0, \sigma^2)$. The standard deviation σ of this normal distribution represents the characteristic inter-species interaction strength. To ensure the stability of the system, the diagonal elements of $\mathbf{A}$ are set to be $a_{ii} = -dC$ in tuning C or $a_{ii} = -d\sigma$ in tuning σ. Here d is a positive constant. All other entries of $\mathbf{A}$ are set to be zero.

We generated k source communities, $S_1, S_2, \cdots, S_k$, each with N_s species drawn from a pool of $N = 90$ species. To simplify the MST problem, the intrinsic growth rates of all species were set to be identical ($r = 0.5$). The composition vectors of $S_1, S_2, \cdots, S_k$ (denoted as $\boldsymbol{y}^{(1)}, \boldsymbol{y}^{(2)}, \cdots, \boldsymbol{y}^{(k)}$, respectively) were obtained by running the GLV model (i.e., numerically solving the ordinary differential equations (ODEs) in the GLV model) with initial species abundances randomly chosen from a uniform distribution $\mathcal{U}(0,1)$, until a steady state was reached and then normalizing the steady-state abundance of each species by the total biomass of the community.

The sink obtained by simultaneously mixing the k sources was simulated as follows:

1) The mixing proportion of k sources were randomly drawn from a uniform distribution with constraint $\sum_{a=1}^{k} m_a = 1$.
2) The initial composition of the sink community is calculated as: $\boldsymbol{x}(0) = m_1\boldsymbol{y}^{(1)} + m_2\boldsymbol{y}^{(2)} + \cdots + m_k\boldsymbol{y}^{(k)}$. And the initial (absolute) abundance vector is chosen to be $\boldsymbol{X}(0) = \boldsymbol{x}(0)$.

3) Run the GLV model until it reaches a steady state and normalize the steady-state abundance vector by the total biomass of the sink community to get the final composition of the sink community.

Consider a particular mixing order π among the total $k!$ mixing orders. Let $\pi(a)$ denote the label of the a-th source in the mixing order. $a, \pi(a) \in \{1, \cdots, k\}$. The sink obtained by mixing the k sources in the order π was simulated as follows:

1) The mixing proportions of the k sources were set to be equal: $m = \frac{1}{k}, a = 1, \cdots, k$.
2) The initial abundance vector of the sink community is determined by the composition of the first source in the order π, i.e., $\pi(1)$, as $\boldsymbol{X}_0^{(1)} = m\, \boldsymbol{y}^{(\pi(1))}$. Then we run the GLV model until it reaches a steady state. Denote the steady-state abundance vector as $\boldsymbol{X}_{\text{ss}}^{(1)}$.
3) Then the second source $\pi(2)$ arrives. Right after the mixing, the abundance vector of the sink community becomes $\boldsymbol{X}_0^{(2)} = \boldsymbol{X}_{\text{ss}}^{(1)} + m\, \boldsymbol{y}^{(\pi(2))}$. Then we run the GLV model until it reaches a steady state. Denote the steady-state abundance vector as $\boldsymbol{X}_{\text{ss}}^{(2)}$.
4) Repeat step-3 until all the k sources have been added to the sink. Note that right after the arrival of the k-th source, the abundance vector of the sink community becomes $\boldsymbol{X}_0^{(k)} = \boldsymbol{X}_{\text{ss}}^{(k-1)} + m\, \boldsymbol{y}^{(\pi(k))}$. Then we run the GLV model until it reaches a steady state. Denote the steady-state abundance vector as $\boldsymbol{X}_{\text{ss}}^{(k)}$.
5) Normalize the final steady-state abundance vector $\boldsymbol{X}_{\text{ss}}^{(k)}$ by the total biomass of the sink community to get the final composition of the sink community.

Since the input data of MST solvers is the OTU count table, for both sink and source communities, we converted the species relative abundances into counts by multiplying the absolute abundances and a fix number (1,000 in all the simulations) and rounding to the nearest integers as the synthetic count data generated by the GLV model.

2. Microbial interactions affect the assembly of the sink community.

The deep reason why the existing MST solvers are almost doomed to fail in the presence of microbial interactions is that the true contributions of different sources are only reflected in the sink's initial composition, which will evolve to a final composition following complex ecological dynamics. In general, the final composition will be quite different from the initial one. Here we sketch a proof.

Let us consider a sink generated by mixing K non-overlapping sources with compositions given by $\boldsymbol{y}^{(1)}, \dots, \boldsymbol{y}^{(K)}$, respectively. The initial abundance vector of the sink is denoted as $\boldsymbol{X}(0) = (X_1(0), \dots, X_N(0))$, and its initial composition is given by $\boldsymbol{x}(0) = (x_1(0), \dots, x_N(0))$ with $x_i(0) = X_i(0)/\sum_{i=1}^{N} X_i(0)$ representing the relative abundance of species i. Note that $\boldsymbol{x}(0) = \sum_{\boldsymbol{a}} m_a \, \boldsymbol{y}^{(a)}$. Let's assume the population dynamics of the sink community can be represented by a set of ordinary differential equations:

$$\dot{\boldsymbol{X}} = \boldsymbol{f}(\boldsymbol{X}; \boldsymbol{\theta}), \tag{1}$$

where $\boldsymbol{X}(t) = (X_1(t), \dots, X_N(t))$ represents the abundance vector at time t, $\boldsymbol{f}$ is an unspecified nonlinear function with $\boldsymbol{\theta}$ encoding all the ecological parameters, i.e., intrinsic growth rates, and intra- and inter-species interaction strengths. After a small time-step δt, the abundance vector of the sink can be approximated as $\boldsymbol{X}(\delta t) = \boldsymbol{X}(0) + \delta t \, \boldsymbol{f}(\boldsymbol{X}(0); \boldsymbol{\theta})$. The ratio of relative abundance for any species pair (i, j) in the initial community is $\alpha(0) = \frac{x_i(0)}{x_j(0)} = \frac{X_i(0)}{X_j(0)}$, while after δt the ratio becomes:

$$\alpha(\delta t) = \frac{x_i(\delta t)}{x_j(\delta t)} = \frac{X_i(\delta t)}{X_j(\delta t)} = \frac{X_i(0) + \delta t \, f_i(\boldsymbol{X}(\boldsymbol{0}); \boldsymbol{\theta})}{X_j(0) + \delta t \, f_j(\boldsymbol{X}(\boldsymbol{0}); \boldsymbol{\theta})}. \tag{2}$$

If $X_i(0) = X_j(0)$ and $f_i(\boldsymbol{X}(\boldsymbol{0}); \boldsymbol{\theta}) = f_j(\boldsymbol{X}(\boldsymbol{0}); \boldsymbol{\theta})$, then we have $\alpha(\delta t) = \alpha(0)$. But the condition $X_i(0) = X_j(0)$ is too strong to be true. If $X_i(0) \neq X_j(0)$, but $f_i(\boldsymbol{X}(\boldsymbol{0}); \boldsymbol{\theta}) = X_i(0) g_i(\boldsymbol{\theta})$ and $g_i(\boldsymbol{\theta}) = g_j(\boldsymbol{\theta})$, then we have $\alpha(\delta t) = \alpha(0)$. For a general population dynamics model, this requirement means that there are no inter-species interactions and the intrinsic growth rates of different species are identical, which is also too strong to be true. Hence, in general $\alpha(\delta t) \neq \alpha(0)$, and the final composition of the sink community will be quite different from its initial composition.

3. Priority effects affect the assembly of the sink community.

Consider three source communities S_1, S_2 and S_3. Let's assume species-i is only present in source S_1 and species-j is only present in source S_2. We mix the source communities in 6 different orders but with identical mixing proportions $(\frac{1}{3}, \frac{1}{3}, \frac{1}{3})$. For each mixing order, we assume the arrival time of the three sources are 0, τ, 2τ, respectively, where τ is a constant (and is large enough for the resulting sink community to reach a steady state). Suppose we use the composition of the final sink community taken at 3τ to estimate the contribution of each source. We want to prove that at time $t = 3\tau$, the sink communities resulted from different mixing orders will have different compositions, even in the absence of any microbial interactions. To achieve that, let's compute the ratio between the relative abundance of species-i and that of species-j in the final sink community at time $t = 3\tau$, i.e., $\alpha_{ij}(3\tau) = \frac{x_i(3\tau)}{x_j(3\tau)} = \frac{X_i(3\tau)}{X_j(3\tau)}$.

Consider a particular mixing order $S_1 \to S_2 \to S_3$. In the absence of any inter- or intra-species interactions, species will grow exponentially. Hence, at time $t = 3\tau$, the abundance of a species-i (which is only present in source S_1) is given by: $X_i(3\tau) = m_1 X_i(0) \exp(3\tau r_i)$, where $m_1 = \frac{1}{3}$ is the mixing proportion (contribution) of source S_1, $X_i(0)$ is the initial abundance of species-i in source S_1, r_i is the intrinsic growth rate of species-i. Similarly, at time $t = 3\tau$, the abundance of species-j (which is assumed to be only present in source S_2) is given by: $X_j(3\tau) = m_2 X_j(0) \exp(2\tau r_j)$. So, we have

$$\alpha_{ij}^{123}(3\tau) = \frac{m_1 X_i(0) e^{3\tau r_i}}{m_2 X_j(0) e^{2\tau r_j}} = \alpha_{ij}(0) e^{\tau(3r_i - 2r_j)},$$

where the superscript '123' indicates the mixing order $S_1 \to S_2 \to S_3$. We can repeat the above calculation for different mixing orders. The results are summarized here:

$$\alpha_{ij}^{132}(3\tau) = \frac{m_1 X_i(0) e^{3\tau r_i}}{m_2 X_j(0) e^{\tau r_j}} = \alpha_{ij}(0) e^{\tau(3r_i - r_j)},$$

$$\alpha_{ij}^{213}(3\tau) = \frac{m_1 X_i(0) e^{2\tau r_i}}{m_2 X_j(0) e^{3\tau r_j}} = \alpha_{ij}(0) e^{\tau(2r_i - 3r_j)},$$

$$\alpha_{ij}^{231}(3\tau) = \frac{m_1 X_i(0) e^{\tau r_i}}{m_2 X_j(0) e^{3\tau r_j}} = \alpha_{ij}(0) e^{\tau(r_i - 3r_j)},$$

$$\alpha_{ij}^{312}(3\tau) = \frac{m_1 X_i(0) e^{2\tau r_i}}{m_2 X_j(0) e^{\tau r_j}} = \alpha_{ij}(0) e^{\tau(2r_i - r_j)},$$

$$\alpha_{ij}^{321}(3\tau) = \frac{m_1 X_i(0) e^{\tau r_i}}{m_2 X_j(0) e^{2\tau r_j}} = \alpha_{ij}(0) e^{\tau(r_i - 2r_j)}.$$

Note that if the three sources were mixed simultaneously, then we have

$$\alpha_{ij}^{\text{simultaneous}}(3\tau) = \frac{m_1 X_i(0) e^{3\tau r_i}}{m_2 X_j(0) e^{3\tau r_j}} = \alpha_{ij}(0) e^{3\tau(r_i - r_j)}.$$

Therefore, even in the absence of any microbial interactions, different mixing patterns will result in different final compositions of the sink community, which are also different from that obtained by simultaneous mixing.

References

1. Case, T. J. Illustrated guide to theoretical ecology. *Ecology* **80**, 2848–2848 (1999).
2. Erdos, P. & Rényi, A. On the evolution of random graphs. *Publ Math Inst Hung Acad Sci* **5**, 17–60 (1960).
3. Staley, C. *et al.* Predicting recurrence of Clostridium difficile infection following encapsulated fecal microbiota transplantation. *Microbiome* **6**, 166 (2018).
4. Sharon, G. *et al.* Human Gut Microbiota from Autism Spectrum Disorder Promote Behavioral Symptoms in Mice. *Cell* **177**, 1600-1618.e17 (2019).

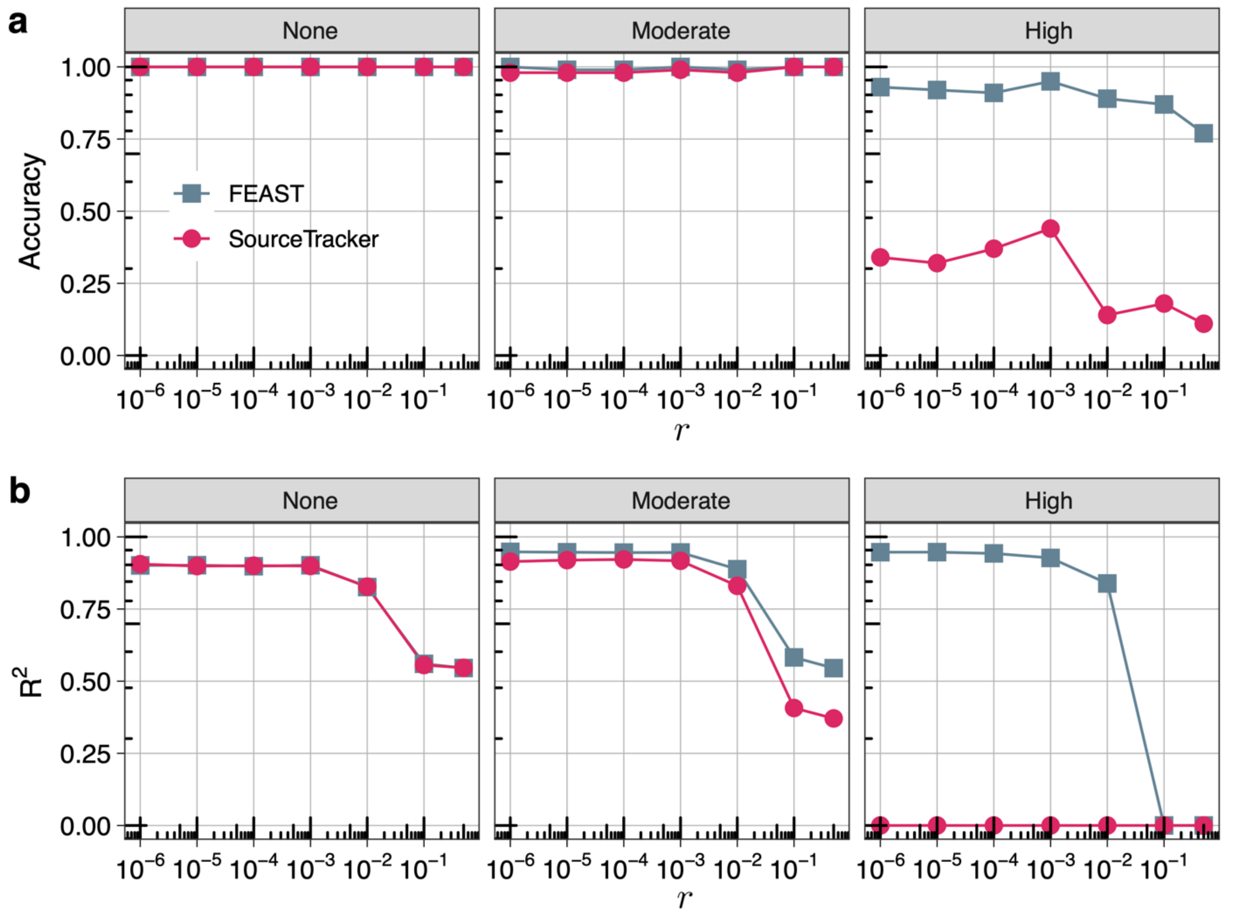

Figure S1: Impact of positive species' mean growth rate on community-based MST. a-b, Performance of SourceTracker (red) and FEAST (blue) evaluated using Accuracy (**a**) and R^2 (**b**) as a function of the species' mean growth rate r for a pool of $N = 90$ species. In all the simulations, we randomly drew the intrinsic growth rate r_i of species-i from a uniform distribution $\mathcal{U}(0, 2r)$ with r the desired mean growth rate (tuned from 10^{-6} to 0.5). For the species pool, we set the network connectivity $C = 0.1$. The diagonal elements of the interaction matrix $\mathbf{A}$ were set to be $a_{ii} = -5C$ to ensure the stability of the community, and the characteristic interaction strength was set to be $\sigma = 0.01$. Each column (from left to right) represents a particular level of species overlap between 10 sources: **None** (each of the 10 sources consists of a unique collection of 9 species chosen from the species pool of size $N = 90$, so any two sources have no species overlap); **Moderate** overlap (for each source, we randomly drew 20 species from the species pool); **High** overlap (for each source, we randomly drew 50 species from the species pool). Among the 10 sources, we randomly selected 3 sources ("true sources") and mixed them with random proportions $(m_1, m_2, m_3 = 1 - m_1 - m_2)$ to form a sink. We repeated this 100 times to form 100 sinks. Accuracy is defined as the fraction of those 100 sinks whose true sources were all correctly predicted (i.e., among all the 10 possible sources, the three sources with the highest predicted

contributions are the true sources). R^2 is the coefficient of determination between the estimated source contributions and true source contributions.

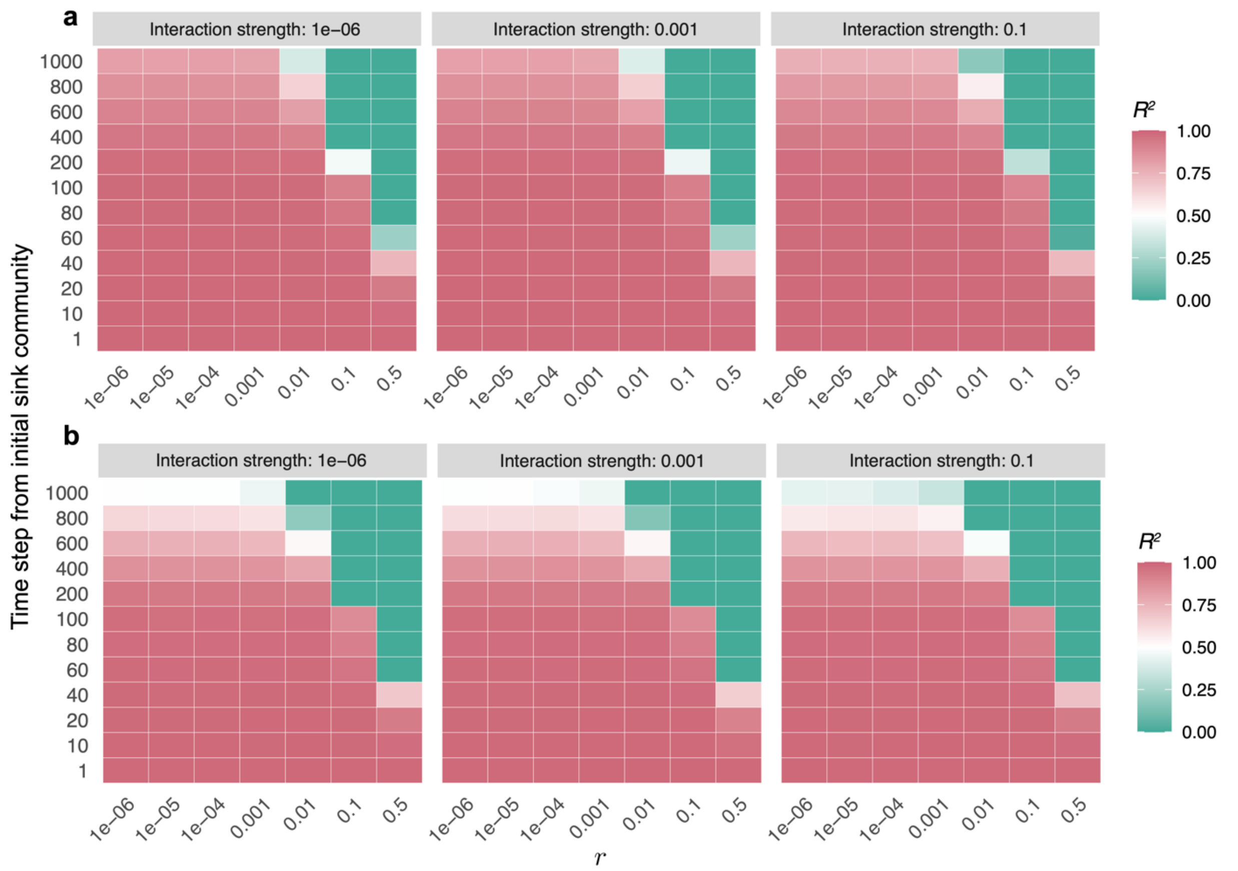

Figure S2: Impact of time scale on the FEAST. a-b, Performance of FEAST evaluated using R^2 as functions of time step from the initial sink community and the species' mean growth rate r for a pool of $N = 90$ species. In all the simulations, we randomly drew the intrinsic growth rate r_i of species-i from a uniform distribution $\mathcal{U}(0, 2r)$ with r the desired mean growth rate (tuned from 10^{-6} to 0.5). For the species pool, we set the network connectivity $C = 0.1$ (a) and 0.3 (b). The diagonal elements of the interaction matrix **A** were set to be $a_{ii} = -5C$ to ensure the stability of the community. Each column (from left to right) represents a particular characteristic interaction strength. Each of the 3 sources consists of a unique collection of 30 species chosen from the species pool of size $N = 90$, so any two sources have no species overlap. We first generated 100 initial sinks by mixing 3 sources with random proportion, then the community of each source will evolve along time. We used the sink communities at different time steps into the source tracking problem. R^2 is the coefficient of determination between the estimated source contributions and true source contributions.

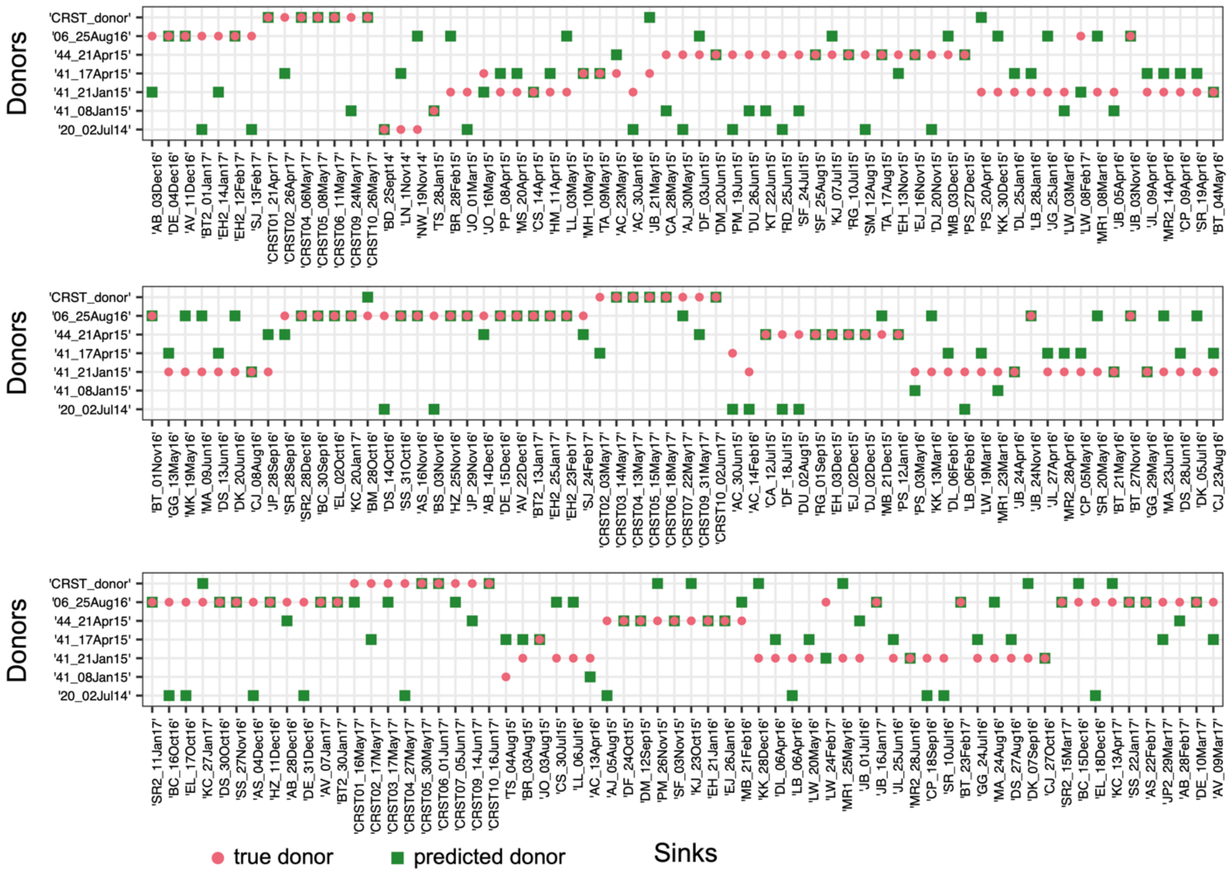

Figure S3: Evaluation of FEAST using FMT data from Staley et al.[3] True donor (red cycle) vs. predicted donor (green square) of each recipient. For each post-FMT community (sink), among all the 7 donors, we referred to the one whose fecal sample has the highest contribution estimated by FEAST as the "predicted donor". In Fig.4c, we presented results of the first 65 sinks. Here, we showed the results of the remaining 194 sinks. Sources: microbiome samples of donors and the pre-FMT samples of recipients; Sinks: post-FMT samples of recipients.

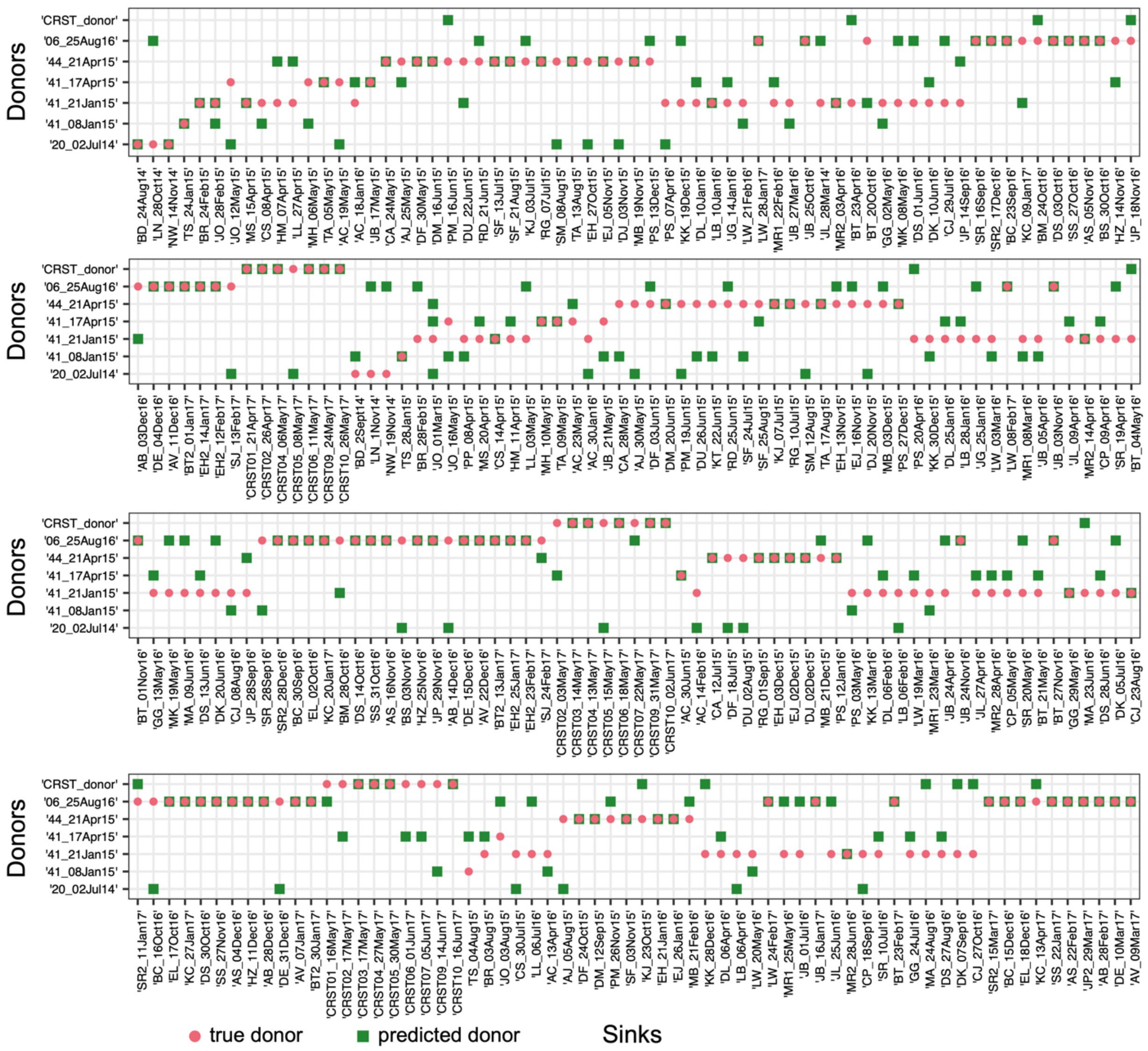

Figure S4: Evaluation of SourceTracker using FMT data from Staley et al.[3] True donor (red cycle) vs. predicted donor (green square) of each recipient. For each post-FMT community (sink), among all the 7 donors, we referred to the one whose fecal sample has the highest contribution estimated by SourceTracker as the "predicted donor". Sources: microbiome samples of donors and the pre-FMT samples of recipients; Sinks: post-FMT samples of recipients.

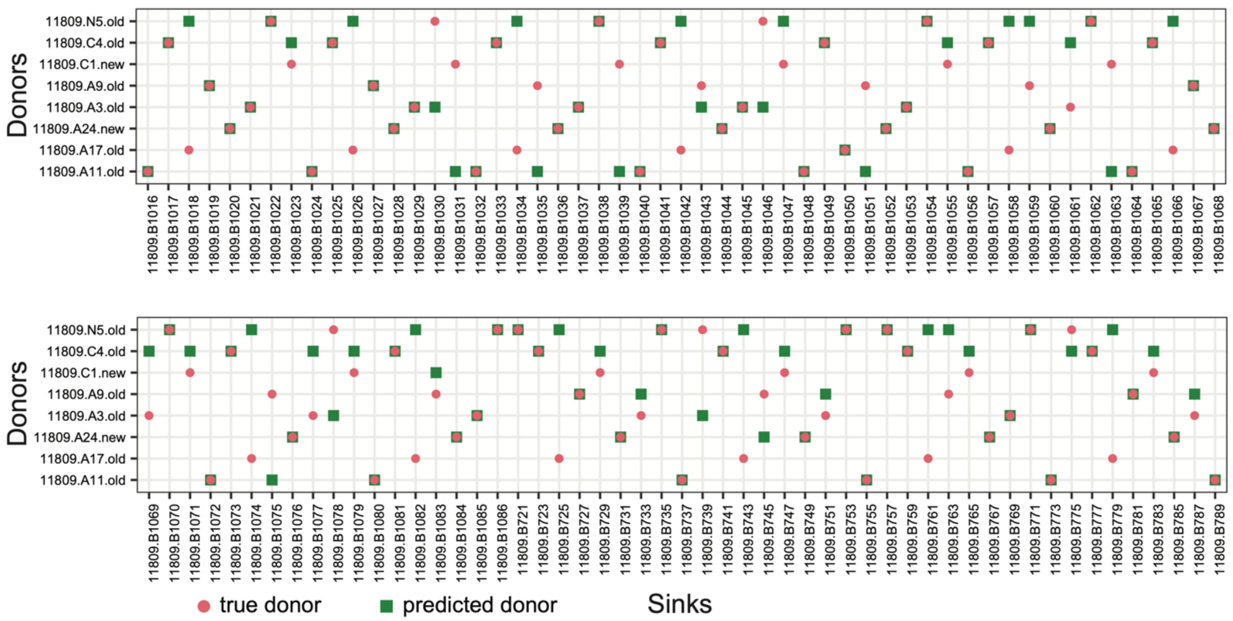

Figure S5: Evaluation of FEAST using FMT data from Sharon et al.[4] True donors (red cycle) vs. the predicted donor (green square) of each recipient sink given by FEAST using the source and sink compositions as the input. For each post-FMT community (sink), among all the 8 donors, we referred to the one whose fecal sample has the highest contribution estimated by FEAST as the "predicted donor". Sources: microbiome samples of donors and the pre-FMT samples of recipients; Sinks: post-FMT samples of recipients. In total, there are 106 sinks.

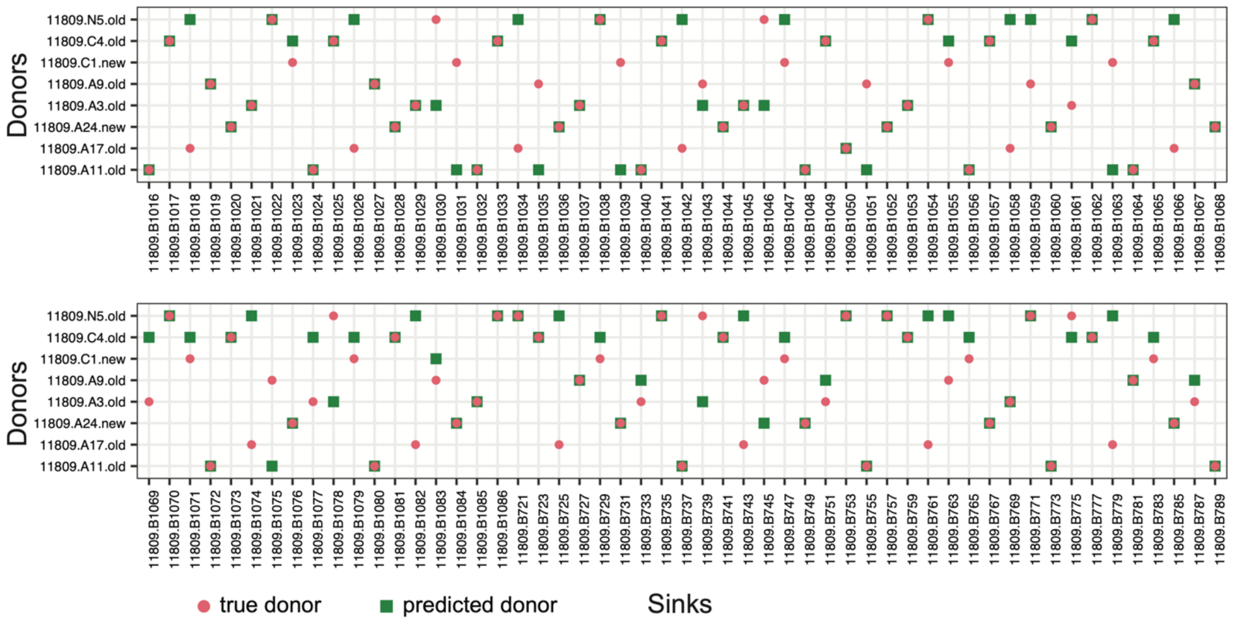

Figure S6: Evaluation of SourceTracker using FMT data from Sharon et al.[4] True donors (red cycle) vs. the predicted donor (green square) of each recipient sink given by SourceTracker using the source and sink compositions as the input. For each post-FMT community (sink), among all the 8 donors, we referred to the one whose fecal sample has the highest contribution estimated by SourceTracker as the "predicted donor". Sources: microbiome samples of donors and the pre-FMT samples of recipients; Sinks: post-FMT samples of recipients. In total, there are 106 sinks.

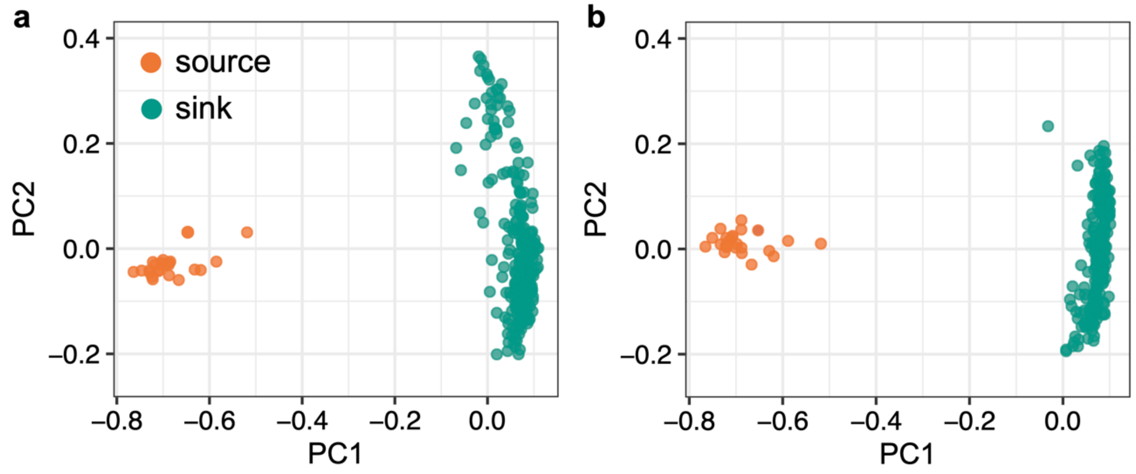

Figure S7: Principal Coordinate Analysis (PCoA) plot of the sinks and sources in the community coalescence experiments. **a**, Pairwise mixing. **b**, Quadruple-wise mixing.

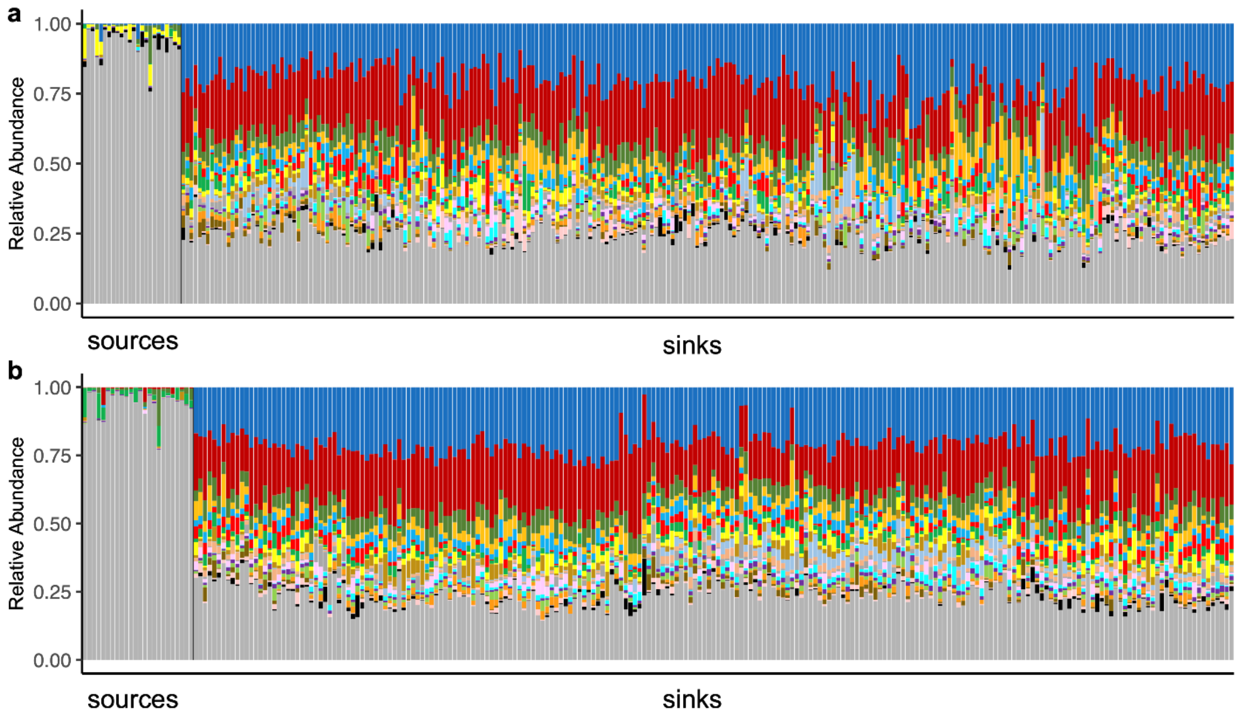

Figure S8: Taxonomic profiles of sources and sinks in community coalescence experiments. **a**, Pairwise mixing. **b**, Quadruple-wise mixing. For visualization purpose, we only showed the top-20 abundant ASVs. All other ASVs were grouped together and shown in gray. We found that some highly abundant ASVs in the source communities have very low abundances in the sink communities, whereas some low-abundance ASVs in source communities flourish in the sink communities. Also, a few ASVs in the sinks were not detected in the sources, indicating that either their relative abundances were below the detection limit or there was potential contamination.

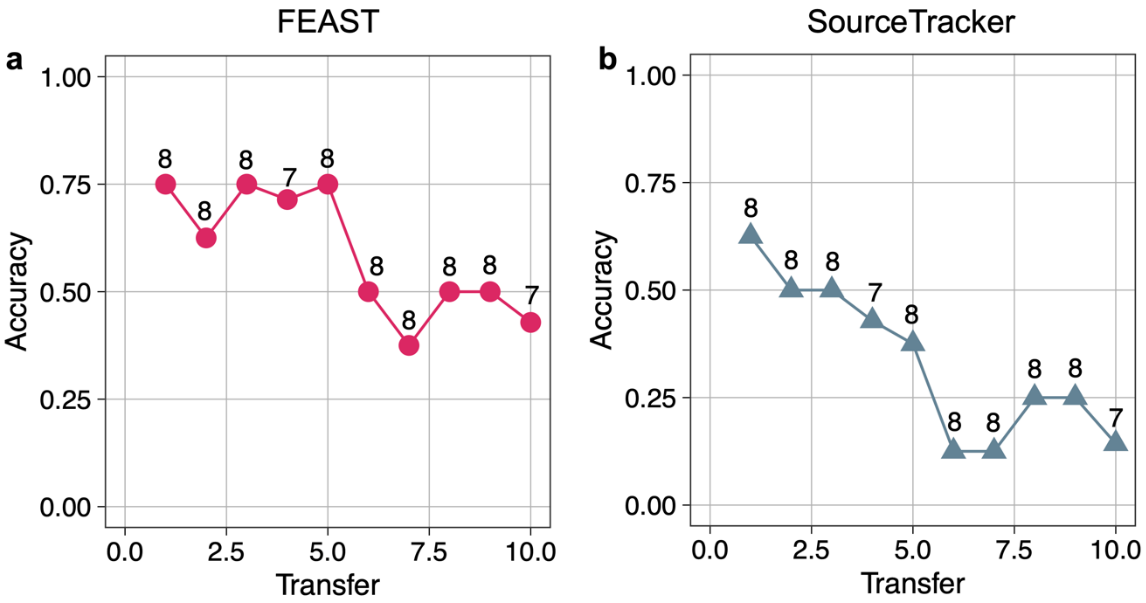

Figure S9: Evaluation of community-based MST solvers in non-mixing dataset. The samples of 8 subjects were transferred in 10 days. We considered the 8 samples collected at day 0 as sources and the samples collected at each transfer as sinks. Accuracy is defined as the fraction of those sinks (number around each point) whose true sources (sample at day 0) were correctly predicted (i.e., among all the 8 possible sources, the source with the highest predicted contributions is the true sources). The number represents the total number of sinks.

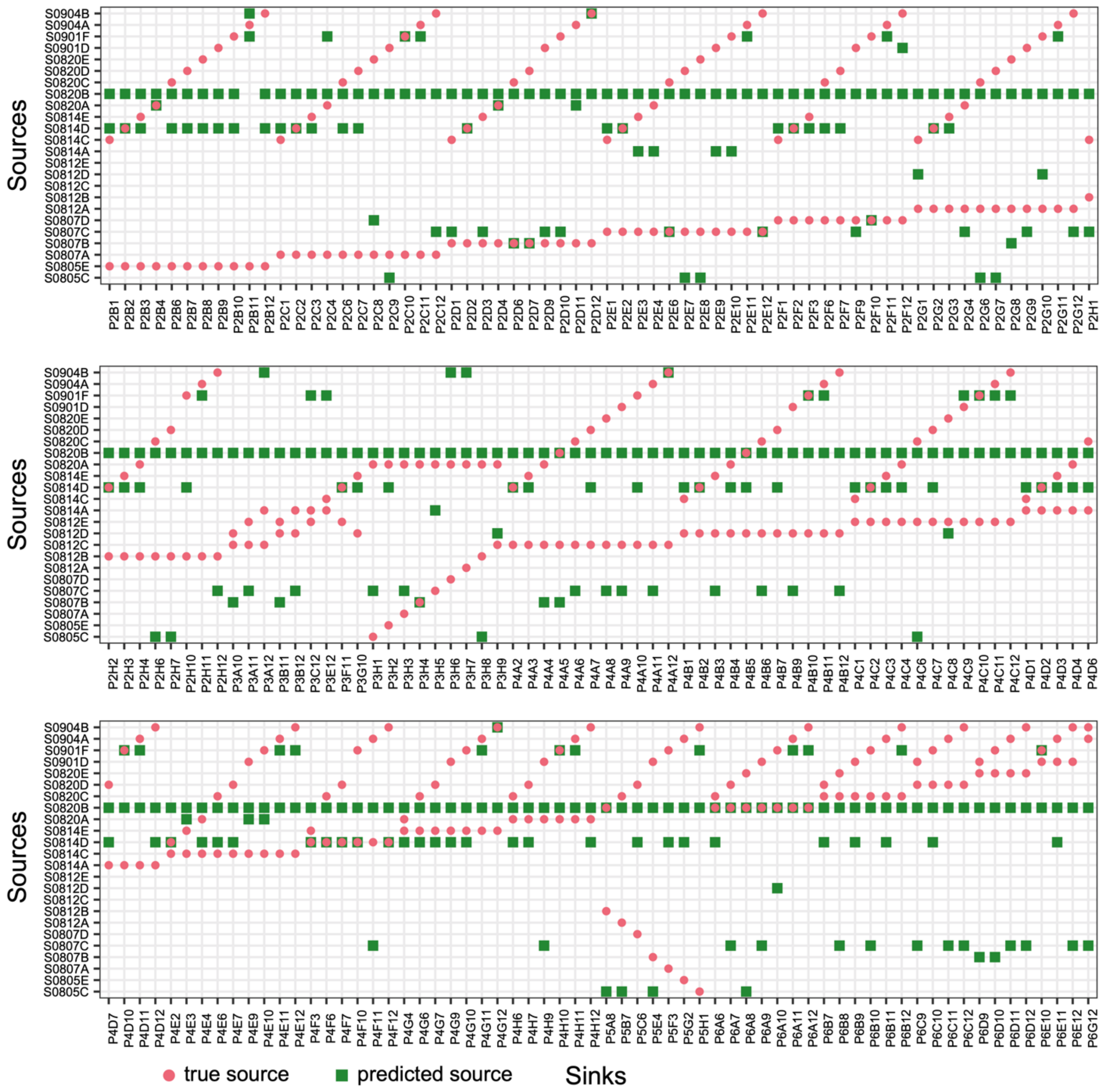

Figure S10: Performance of FEAST in identifying sources in pairwise community coalescence experiments. True sources (red cycles) vs. predicted sources (green squares) of each sink. For each sink, among the 24 known sources, the two sources with the top-two largest contributions predicted by FEAST were referred to as the predicted sources. In Fig.5, we showed the results of the first 64 sinks. Here we showed the results of the remaining 192 sinks.

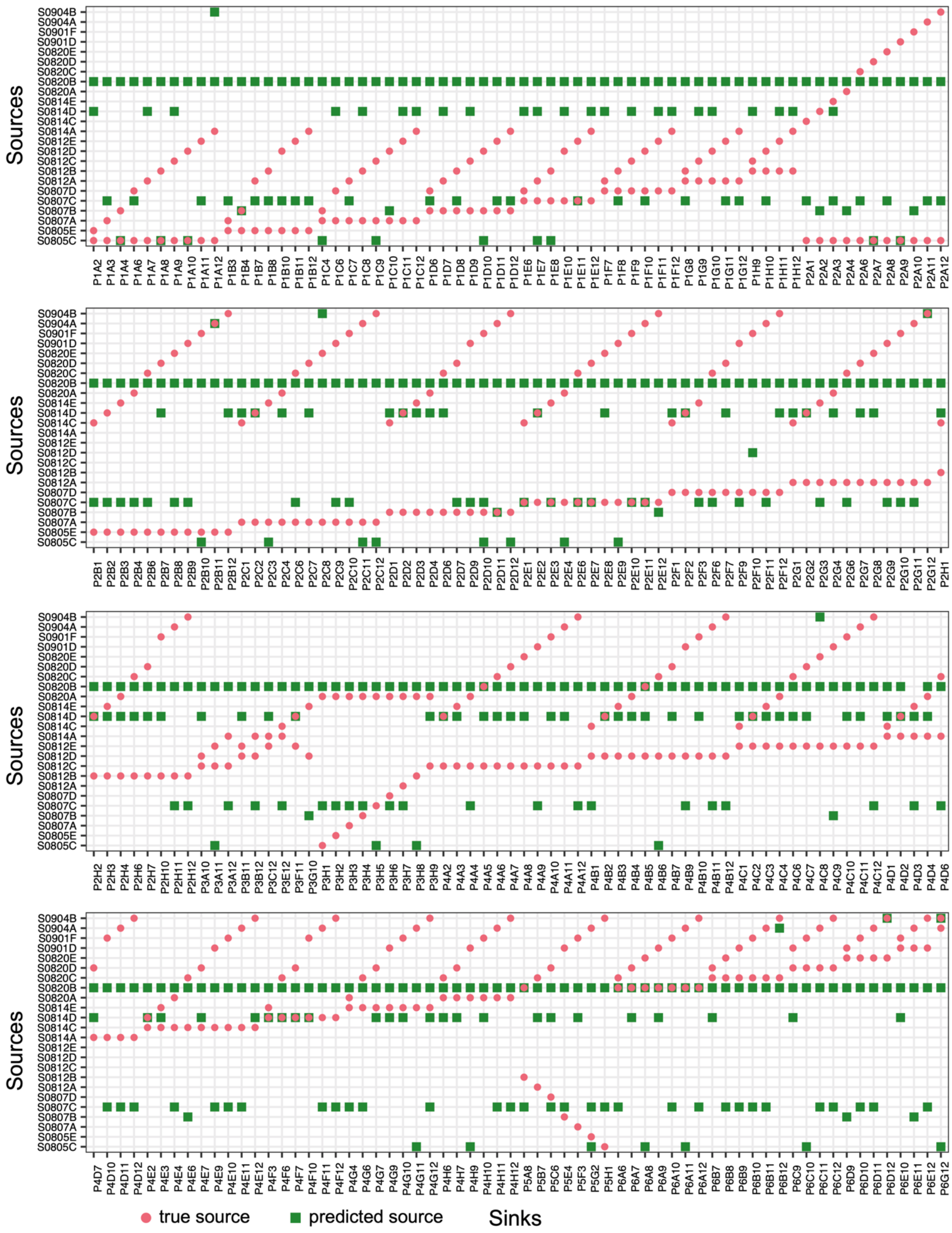

Figure S11: Performance of SourceTracker in identifying sources in pairwise community coalescence experiments. True sources (red cycles) vs. predicted sources (green squares) of each sink. For each sink, among the 24 known sources, the two sources with the top-two largest contributions predicted by SourceTracker were referred to as the predicted sources.

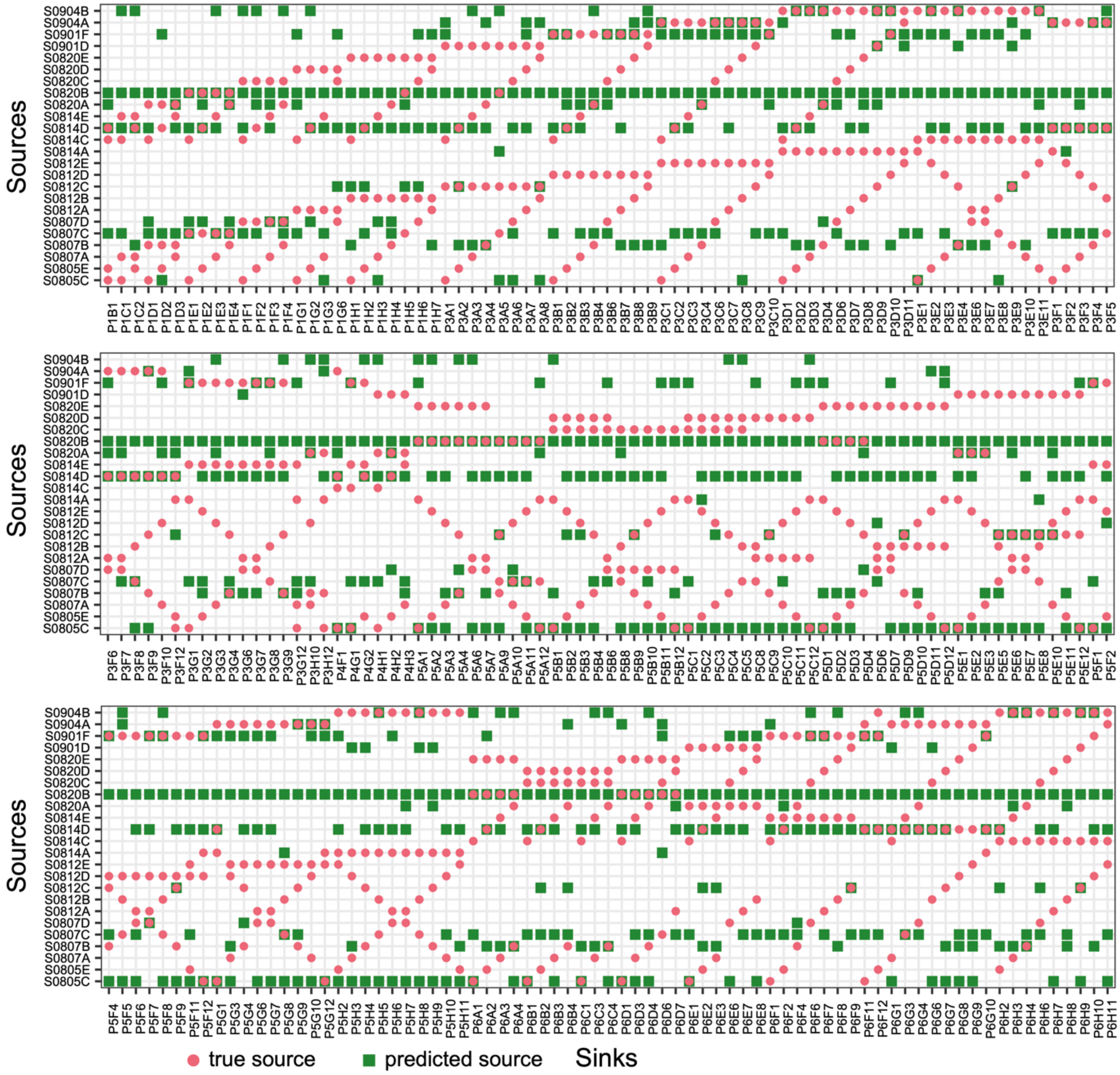

Figure S12: Performance of FEAST in identifying sources in quadruple-wise community coalescence experiments. There are 24 source communities (stool samples from 24 healthy individuals). Each sink community is obtained by mixing four different source communities *ex vivo*. The final composition of each sink was obtained from metagenomic sequencing of samples collected after 11 days of the *ex vivo* mixing. True sources (red cycles) vs. predicted sources (green squares) of each sink. (Each row includes 75 sinks). For each sink, among the 24 known sources, the four sources with the top-four largest contributions predicted by FEAST were referred to as the predicted sources.

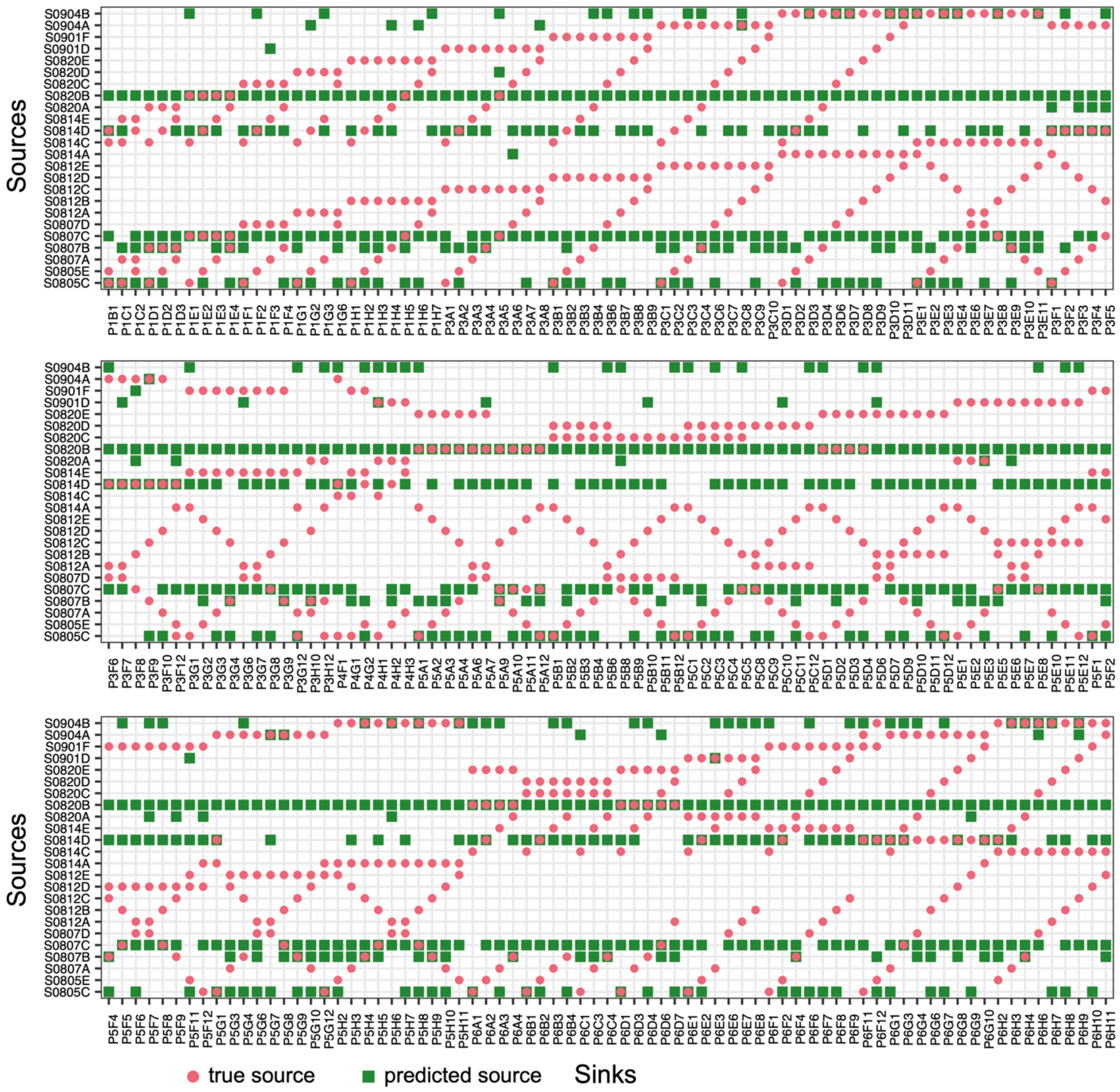

Figure S13: Performance of SourceTracker in identifying sources in quadruple-wise community coalescence experiments. There are 24 sources communities (stool samples from 24 healthy individuals). Each sink community is obtained by mixing four different source communities *ex vivo*. The final composition of each sink was obtained from metagenomics sequencing of samples collected after 11 days of the *ex vivo* mixing. True sources (red cycles) vs. predicted sources (green squares) of each sink. (Each row includes 75 sinks). For each sink, among the 24 known sources, the four sources with the top-four largest contributions predicted by SourceTracker were referred to as the predicted sources.

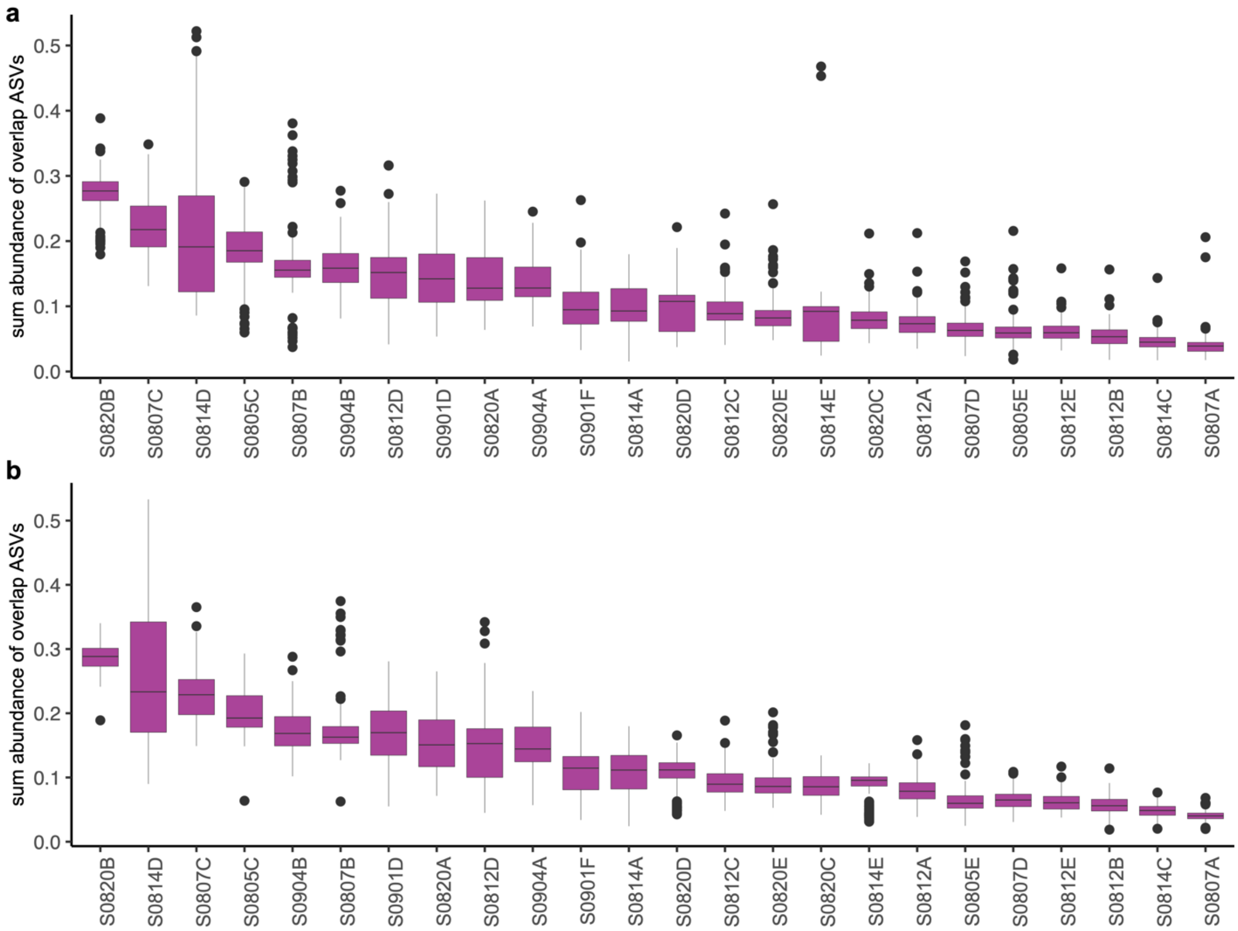

Figure S14: Relative abundances of common ASVs shared by sources and sinks. For each sink and source pair, we identified their common ASVs and calculated the total relative abundance of those common ASVs. Each boxplot represents the total relative abundance of common ASVs shared by this source and each of the 256 sinks in the pairwise community coalescence experiments (**a**); and 225 sinks in quadruple-wise community coalescence experiments (**b**).
